# The smooth muscle titin filament system regulates uterus contractility and implantation

**DOI:** 10.1016/j.isci.2026.117888

**Published:** 2026-10-14

**Authors:** V Juhi Sur, Claudia Fink, Nora Bergmann, Rene Jüttner, Melissa Klingeberg, Fabian Coscia, Lorena Suarez-Artiles, Philipp Mertins, Johanna Elisabeth Fritsch, Melissa Birol, Maik Gollasch, Michael Gotthardt

**Affiliations:** 1 Translational Cardiology and Functional Genomics, Max Delbrück Center for Molecular Medicine, 13125 Berlin, Germany; 2 DZHK (German Centre for Cardiovascular Research), Partner Site Berlin, Berlin, Germany; 3 Spatial Proteomics, Max Delbrück Center for Molecular Medicine, 13125 Berlin, Germany; 4 Humboldt University of Berlin, Faculty of Life Science, Berlin, Germany; 5 Proteomics Platform, Max Delbrück Center for Molecular Medicine in the Helmholtz Association, 13125 Berlin, Germany; 6 Single Molecule Biophysics Probing Quantitative Neuroscience, Berlin Institute for Medical Systems Biology, 10115 Berlin, Germany; 7 Experimental and Clinical Research Center (ECRC), Charité Medical Faculty and Max Delbrück Center for Molecular Medicine in the Helmholtz Association (MDC), 13125 Berlin, Germany; 8 Geriatric Medicine, University Medicine Greifswald, 17475 Greifswald, Germany; 9 Cardiology, Virchow Klinikum, Charité - University Medicine, 13353 Berlin, Germany; 10 Charité Universitätsmedizin Berlin, corporate member of Freie Universität Berlin and Humboldt-Universität zu Berlin, Charitéplatz 1, 10117 Berlin, Germany

**Keywords:** uterus, implantation, titin, NBR1, p62, biomechanics, smooth muscle, ROS, calcium, pregnancy, mouse models

## Abstract

Coordinated uterine peristalsis positions embryos for implantation, but the mechanotransduction pathways controlling uterine wave coordination are not well defined. We combined smooth-muscle-specific deletion of titin M-band exons 1 and 2 with vertical agarose gel electrophoresis, mass spectrometry, uterine ring myography, imaging, transcriptomics, spatial proteomics, and targeted immunoblotting. We show that the myometrium expresses smooth-muscle titin and low-abundance full-length titin including the M-band kinase region. Conditional M-band deletion increased oxytocin-evoked force and area-under-the-curve while reducing coordinated wall motion, yielding fewer as well as crowded/mislocalized implantation sites and heavier placentas. Proteomics and immunoblotting identified disruption of an NBR1/p62 autophagy hub with downstream remodeling of excitability, metabolism, and wall mechanics. Reduced telokin and MLCK levels are consistent with impaired relaxation and increased Ca^2+^-sensitization. Together, these findings establish the titin M-band as a mechanotransductive signaling hub regulating peri-implantation motility with potential clinical relevance for optimizing implantation after *in vitro* fertilization and preventing ectopic pregnancy.

## Highlights

• The myometrium expresses full-length titin with the M-band kinase region
• Titin M-band loss increases force but disrupts uterine coordination
• NBR1/p62 proteostasis and redox pathways are altered in KO myometrium
• Ion handling and matrix coupling changes link titin loss to embryo crowding

## Graphical abstract

**Figure:**
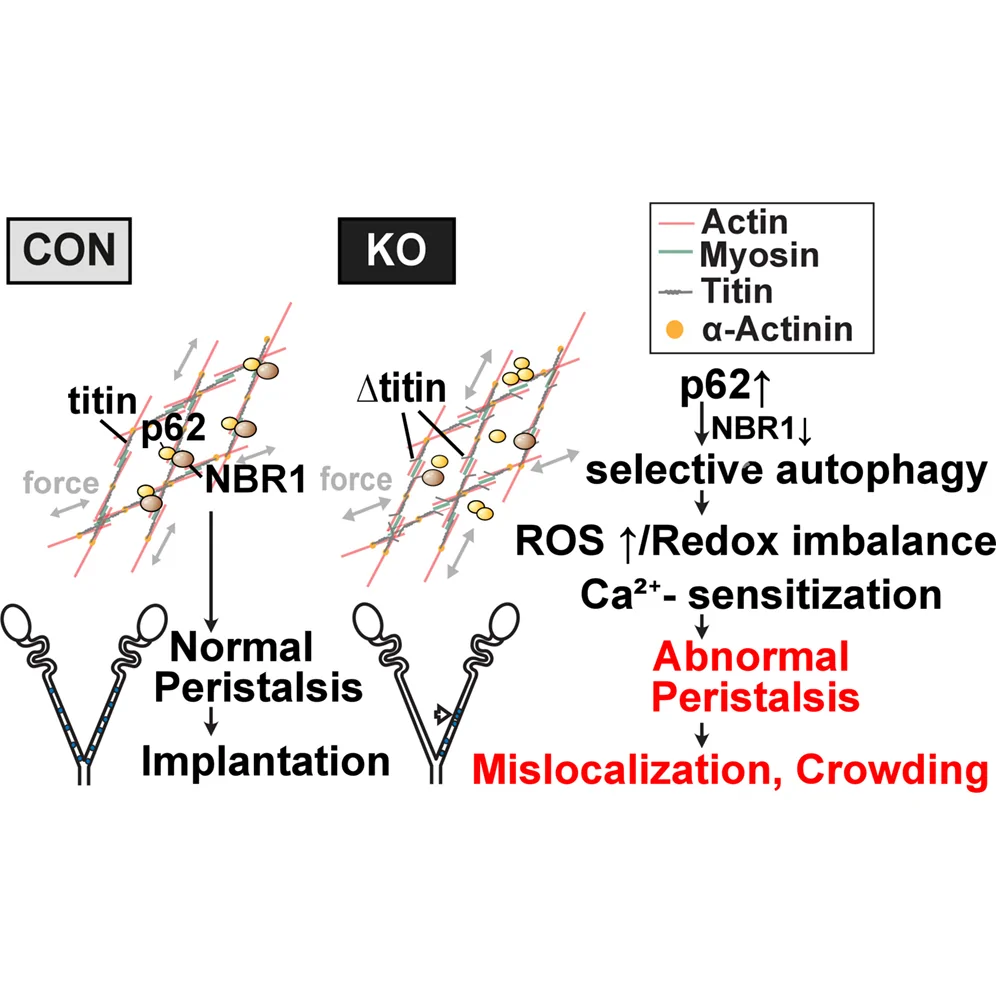

## Teaser

Human Physiology; Cellular physiology; Cell biology

## Introduction

Human infertility is a global problem and in the United States approximately 12% of women have difficulties getting pregnant or carrying a baby to term.^1^ Conception can be improved by *in vitro* fertilization and embryo transfer techniques. Nevertheless, implantation failure and pregnancy loss are not only common problems after assisted reproduction, but also after natural conception. Moreover, improper embryo placement can lead to ectopic pregnancies that endanger fetal as well as maternal health or mislocalization within the uterus that results in obstruction of the birth canal (placenta previa). The mechanistic details of smooth muscle contraction and relaxation that contribute to the localization of the embryo within the uterus are still unclear: In contrast to striated muscle with well-characterized sarcomeres, the structure, function, and signaling pathways of smooth muscle contractile units are only incompletely understood.

Titin is a giant protein and abundant in striated muscle of vertebrates.^2^^,^^3^ It connects the Z-disc and M-band of the sarcomere, determines its elastic properties and has been identified as the most common cause of inherited heart disease.^4^^,^^5^^,^^6^^,^^7^ Its role in mechanosignaling relates to the kinase region, which interacts with calmodulin and the autophagy adaptor p62/Sequestosome 1 to regulate striated muscle function and growth.^8^^,^^9^

Smooth muscle titin (Smitin) was first described in chicken gizzard 40 years ago,^10^ followed by the detection of a 0.9 MDa isoform^11^ that interacts *in vitro* with both smooth muscle α-actinin^12^ and smooth muscle myosin.^11^^,^^13^ The first description of smooth muscle titin transcripts in rabbit uterus^14^ was followed by the identification of smooth muscle titin in human carotid artery, bladder, and uterus—although at levels up to 100-fold lower than in striated muscle.^15^ Although several labs have validated the expression of a ∼1 MDa smooth muscle titin isoform, there is conflicting information on the expression of a full-length titin and how titin relates to the elastic and contractile properties of smooth muscle. Recent work has shown that titin regulates arterial stiffness through vascular smooth muscle cell tone in male rats, where RBM20 knockout produced a larger and more compliant titin isoform, while information on non-vascular smooth muscle is lacking.^16^

Building on these findings, we define titin’s role in the uterus using a smooth muscle-specific titin knockout mouse targeting C-terminal M-band exons encoding the kinase region. We show that the myometrium expresses not only smitin but also a low-abundance full-length titin isoform containing the kinase domain. Loss of this region preserved basal uterine morphology yet produced a striking phenotype: unlike skeletal muscle, where titin strongly contributes to the elastic properties, uterine M-band deletion primarily altered active contractility, increasing force generation while reducing coordinated wall motion, resulting in impaired implantation with abnormal site distribution, including embryo crowding and mislocalization. Proteomics revealed that removal of M-band exons destabilized a selective autophagy complex, with NBR1 reduced while p62/SQSTM1 increased, and MuRF1 remained unchanged. The altered proteostasis state was accompanied by changes in calcium handling, extracellular matrix remodeling, and reduced expression of relaxation-associated proteins. These findings position the titin M-band as a mechanotransductive hub that restrains excitability and synchronizes peristalsis, thereby ensuring correct embryo spacing. By linking a defined structural element to altered implantation dynamics, our work highlights titin as a previously unrecognized regulator of reproductive success and a potential target to optimize implantation in assisted reproduction.

## Results

### The uterus expresses truncated smooth muscle titin and a full-length titin isoform that contains the C-terminal kinase region

Striated muscle titin with up to 4 MDa isoforms requires low-resolution gels for separation,^17^ while smooth muscle titin (alias “smitin”)^13^ is amenable to conventional polyacrylamide gel electrophoresis (PAGE) due to its smaller size. However, expression is up to 100-fold lower than in skeletal muscle.^15^ Conflicting information on smooth muscle titin expression, with only 1/3 of the >300 exons included in the mature transcript and uterus titin only detected with antibody staining against Z-4-6 and Ig34-36,^11^^,^^12^^,^^13^^,^^15^^,^^18^ prompted us to revisit titin expression in striated versus smooth muscle.

We quantified titin mRNA and protein levels and composition in mouse uterus and confirmed expression of a 1 MDa isoform as well as mRNA and protein fragments corresponding to Z-disc, I-band, and M-band exons (Figures 1A–1C and S1). The full-length protein is visible on the agarose gel after extensive overloading with uterus lysate (Figure 1B). We confirmed the identity of the protein by mass spectrometry and found peptide coverage along the complete titin amino acid sequence up to the M-band exons, confirming the existence of a full-length titin (Figure 1C and Table S1). Signal intensities were reduced as compared to gastrocnemius, consistent with lower expression levels.

**Figure 1:**
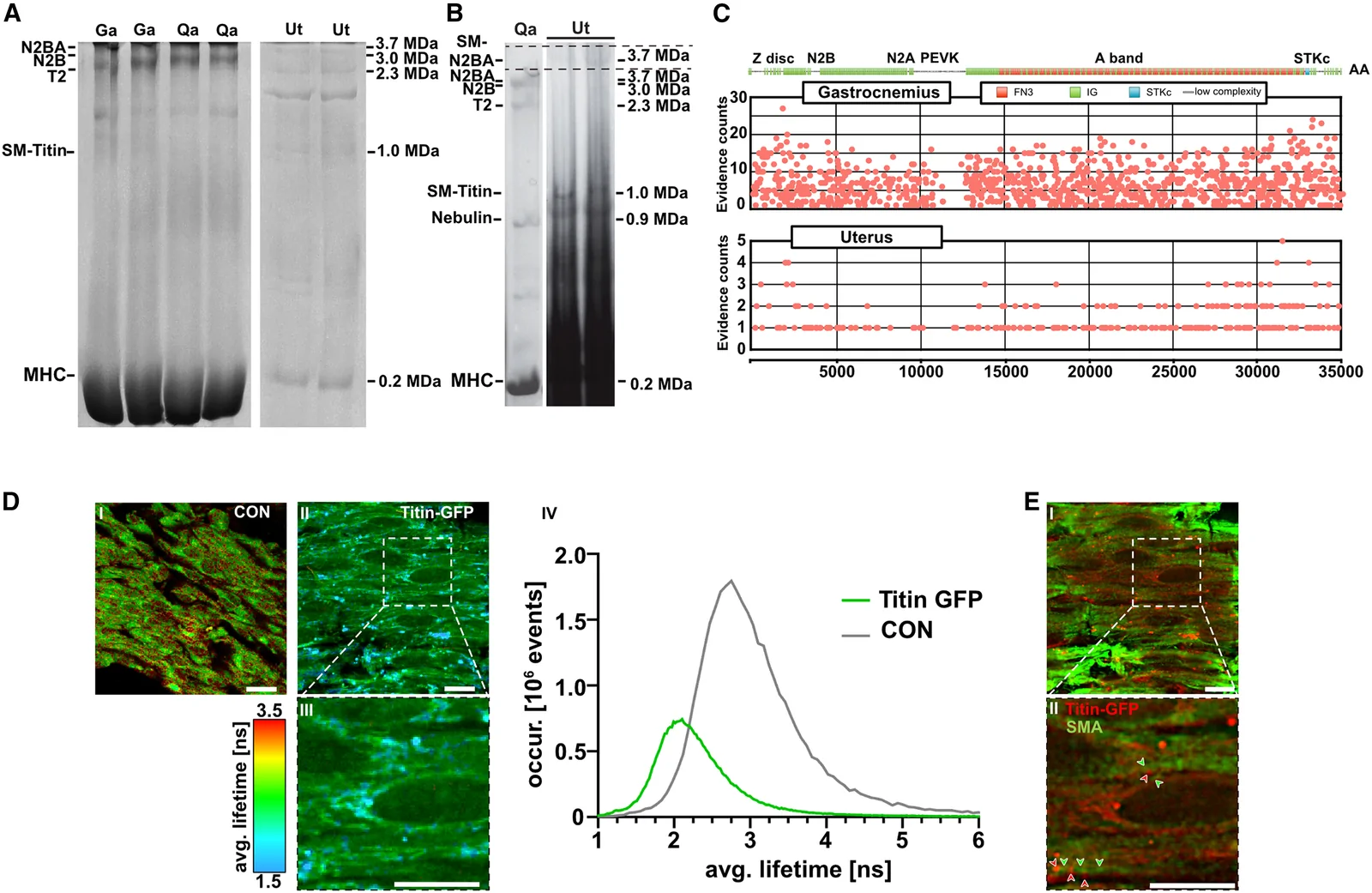
Full-length titin expression and localization in uterine smooth muscle cells (A) SDS gel electrophoresis of Gastrocnemius (Ga), Quadriceps (Qa) with SM-N2BA, N2B, T2, and short smooth muscle titin isoform (SM-Titin) in the uterus. (B) Overloading of uterus lysate on an agarose gel revealed the full-length titin. (C) Identification of titin peptides by targeted mass spectrometry of bands observed on SDS-PAGE. The detected peptides extended throughout the C-terminus of the full-length titin isoform. (D) Fluorescence lifetime imaging of uterus tissue from knock-in mice expressing GFP at the C-terminus of titin shows a fluorescence lifetime of 2.2 ns, compared to >3 ns in controls (green vs. gray line in IV). Scale bars, 10 μm. (E) In uterine tissue, smooth muscle actin (SMA) does not colocalize with titin-GFP, but signals alternate. Scale bars, 10 μm.

Since smitin has been shown to interact with both smooth muscle α-actinin and smooth muscle myosin *in vitro,*^11^^,^^12^^,^^13^ it is presumed to connect *dense bodies* and thick filaments to provide elasticity and structural integrity. To determine the organization of the titin filament system *in vivo*, we used fluorescence life time imaging (FLIM) of uterine smooth muscle cells isolated from wild-type (WT) and GFP-knockin mice.^19^^,^^20^ The average fluorescence life time of Titin-GFP was 2.3 ns, consistent with the known intrinsic lifetime of GFP, while WT showed a longer mean lifetime (>3 ns) reflecting tissue autofluorescence (Figure 1D). The GFP signal showed a distinct perinuclear and filament-associated distribution. Co-immunostaining for Titin-GFP and smooth muscle actin (SMA) indicates the expression of full-length titin and the C-terminal GFP alternating with the N-terminal with actin filaments of smooth muscle cells, supporting its integration into the cytoskeletal network (Figure 1E). Together, our data reveal that titin is expressed in the uterus, with full-length titin protein levels an order of magnitude lower than smitin levels and integrated into a discrete filament network.

### The SMMHCcre transgene efficiently removes the titin kinase region in smooth muscle, which does not alter basal uterine morphology or function in knockout mice

To determine the role of titin’s kinase region in uterine smooth muscle, we mated the conditional knockout with floxed M-band exon 1 and 2 (MEx1-2)^21^ to the smooth muscle myosin heavy chain (SMMHC) cre transgenic (Cre+).^22^ The recombinase is expressed in smooth muscle, and recombination has been documented in the vascular wall, GI tract and glomeruli of the kidney. To confirm activity in uterine smooth muscle, we mated SMMHCcre mice to GtROSA26 reporter mice^23^ and documented recombination in longitudinal and circular uterine muscle fibers of 90-day-old female double transgenic animals (Figure S1A). We generated experimental animals by exclusively breeding flox heterozygous Cre transgenic male animals to female homozygous floxed animals, as the *Smmhc* promoter is active at low levels in the female germline^24^ (confirmed in Figures S1B and S1C). This breeding scheme produced 25% Cre negative homozygous floxed animals that were used as controls (CON), and 25% Cre-positive homozygous floxed animals, from here referred to as smooth muscle knockout animals (KO). In KO mice, the Cre-recombinase excised titin’s M-band exons 1 and 2 by recombination of the flanking lox-sites (Figure S1B). Analysis of tail and uterine tissue from controls (CON), heterozygous floxed mice (HET), knockout mice (KO), and SMMHCcre animals (Cre+) confirmed the presence of the floxed titin allele (lox), the recombined allele (rec), and the cre transgene (cre) and produced the expected genotypes (Figure S1C).

Expression of titin mRNA after deletion of titin’s M-band exons 1 and 2 in non-pregnant uterus was quantified by quantitative real-time PCR (Figure S1D). We confirmed expression of isoforms that extend into the M-band region, including Z-disc exon 3 (*ZEx3*), the N2A region (*N2A*), and the M-band exon 6 (*MEx6*) in the uterus of control and knockout mice. M-band exons 1 and 2 (*MEx1-2*) were specifically depleted in the smooth muscle knockout.

KO mice did not display any obvious phenotypic abnormalities under baseline conditions, with normal body weight, heart weight, estrous cycle, and uterus histology (Figures S2A–S2D).

### Titin M-band deletion disrupts embryo implantation via altered uterine contractility and embryo positioning

In smooth muscle titin KO mice, ovulation, fertilization, and blastocyst development were normal, but embryo implantation was markedly impaired (Figures 2 and S2).

**Figure 2:**
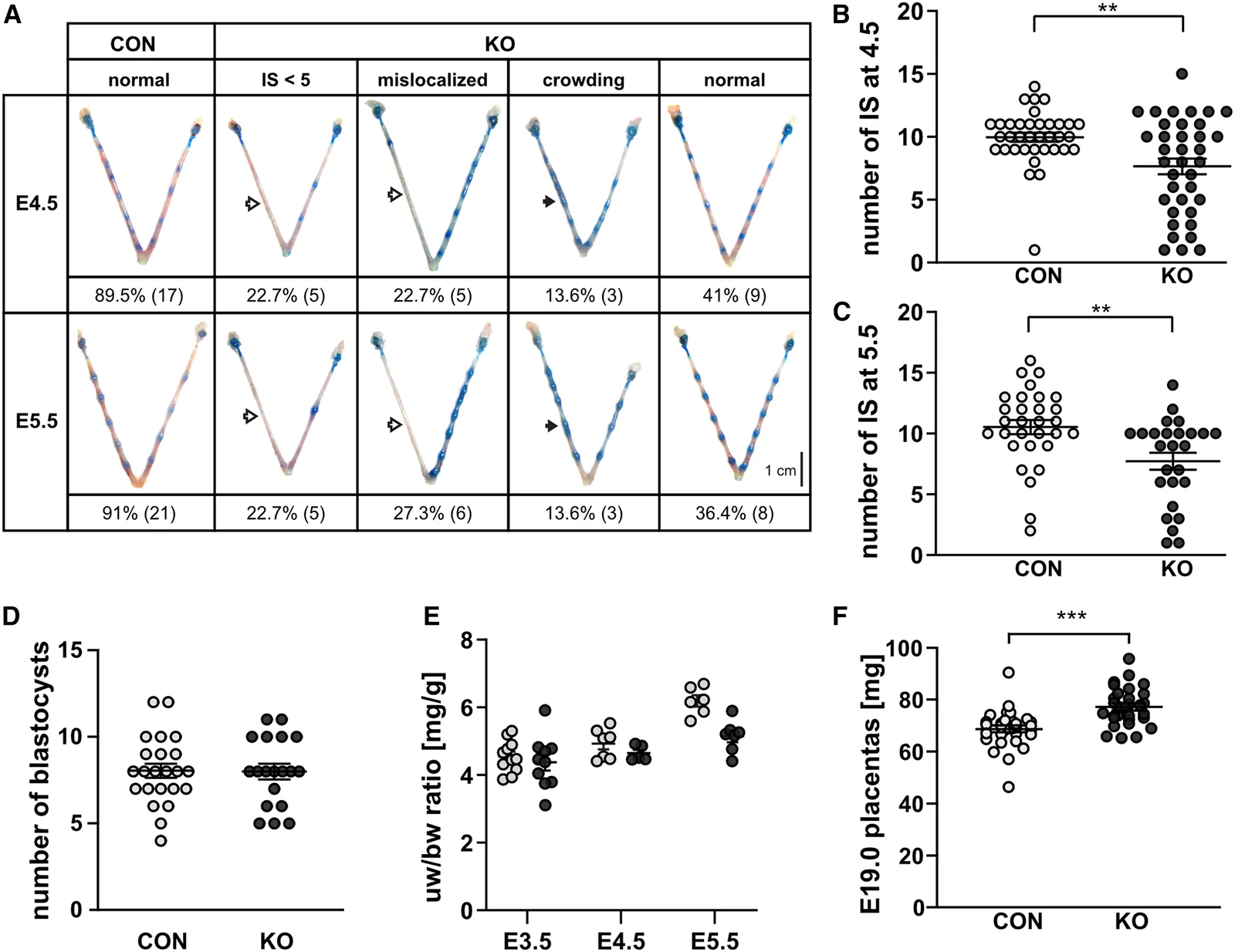
Loss of titin’s kinase region in smooth muscle led to implantation defects and altered uterine development (A) Implantation-site distribution at E4.5 and E5.5. Uteri were classified as normal when Evans Blue-positive implantation sites were distributed along both uterine horns without marked reduction in implantation-site number or visible clustering. KO uteri showed impaired implantation characterized primarily by reduced numbers of implantation sites, with occasional clustering/crowding or abnormal distribution. White arrows indicate uterine regions lacking expected implantation sites; black arrows indicate examples of crowding. (bar 1 cm; absolute numbers of mothers in parentheses). (B and C) Quantification of implantation sites at E4.5 and E5.5 with a significant reduction in KO animals. (D) The total number of blastocysts at E3.5 available for implantation was unchanged between CON (*n* = 23 pregnancies) and KO (*n* = 19). (E) Uterus weight/body weight ratio (uw/bw) was similar in KO and CON animals at E3.5 and E4.5, but did not increase as much in KO uteri at E5.5 (IS = 10; E3.5 KO *n* = 10; CON *n* = 11; E4.5 (KO *n* = 4; CON *n* = 4); E5.5 KO *n* = 6; CON *n* = 6; mean with SEM). (F) Weight of placentas was significantly increased in KO animals at E19. Fisher’s exact test (B, C, and D), two-way ANOVA followed by Bonferroni’s post hoc test was performed to test differences between CON and KO groups∗ *p* < 0.05, ∗∗*p* < 0.01 (E); Student’s *t* test (F), ∗*p* < 0.05, ∗∗*p* < 0.01, ∗∗∗*p* < 0.001. Data are presented as mean ± SEM.

We visualized implantation sites using injection of Evans Blue, which is enriched at regions with increased stromal vascular permeability.^25^ In only ∼40% of KO mice, blastocysts were properly localized at E4.5 and E5.5. The remaining ∼60% had a reduced number of implantation sites (IS; <5), crowding, or mislocalization and a decreased uterus-to-bodyweight ratio at E 5.5 (Figures 2A–2C and S2E–S2G). The reduced number of implantation sites was not the result of a reduced number of blastocysts, which was identical in KO and CON animals (Figure 2D). Consistent with the implantation defect, the increase in uterus weight during pregnancy of CON animals was reduced in KO animals from E5.5 (Figure 2E). In heterozygotes and Cre-positive control animals, uterus to bodyweight ratio and implantation were normal, excluding an effect of the Cre transgene or recombination in the germline (Figures S2E and S2F). At term (E19), placental weight was increased in the KO uterus (Figure 2F), suggesting compensatory placental adaptation to impaired embryo distribution. Average litter size at birth was reduced to less than 50% in KO mice (Figure S2H).

### Deletion of titin’s M-band region is associated with altered mechanosignaling

To identify the molecular basis of the implantation defect, we applied a transcriptomics approach. Using Illumina Microarrays, we analyzed uterus tissue at E3.5 (Figures 3 and S3 and Tables S2–S5). The functional networks assigned were based on genes significantly deregulated at *p* < 0.05 (Figure 3B). Several signaling pathways were affected, including the receptor tyrosine kinase *Ptk7* (most significant) and the *regulator of* *G-protein* *signaling* (*Rgs1*), as well as *Mucosa-associated lymphoid tissue lymphoma translocation protein 1 homolog* (*Malt1*) and *TNFAIP3-interacting protein 1* (*Tnip1*), which link to the Nfκb-pathway. Our global analysis identified several genes related to the Oxytocin signaling pathway, including *Camk2a, Cdkn1a, Eef2k, Itpr2, Myl6, Nfatc1,*and *Rock1* (Table S2) and myometrial relaxation and contraction signaling, with altered transcription of *Camk2a, Gng8, Grk6, Igfbp3, Itpr2,* and *Rgs1* (Table S3).

**Figure 3:**
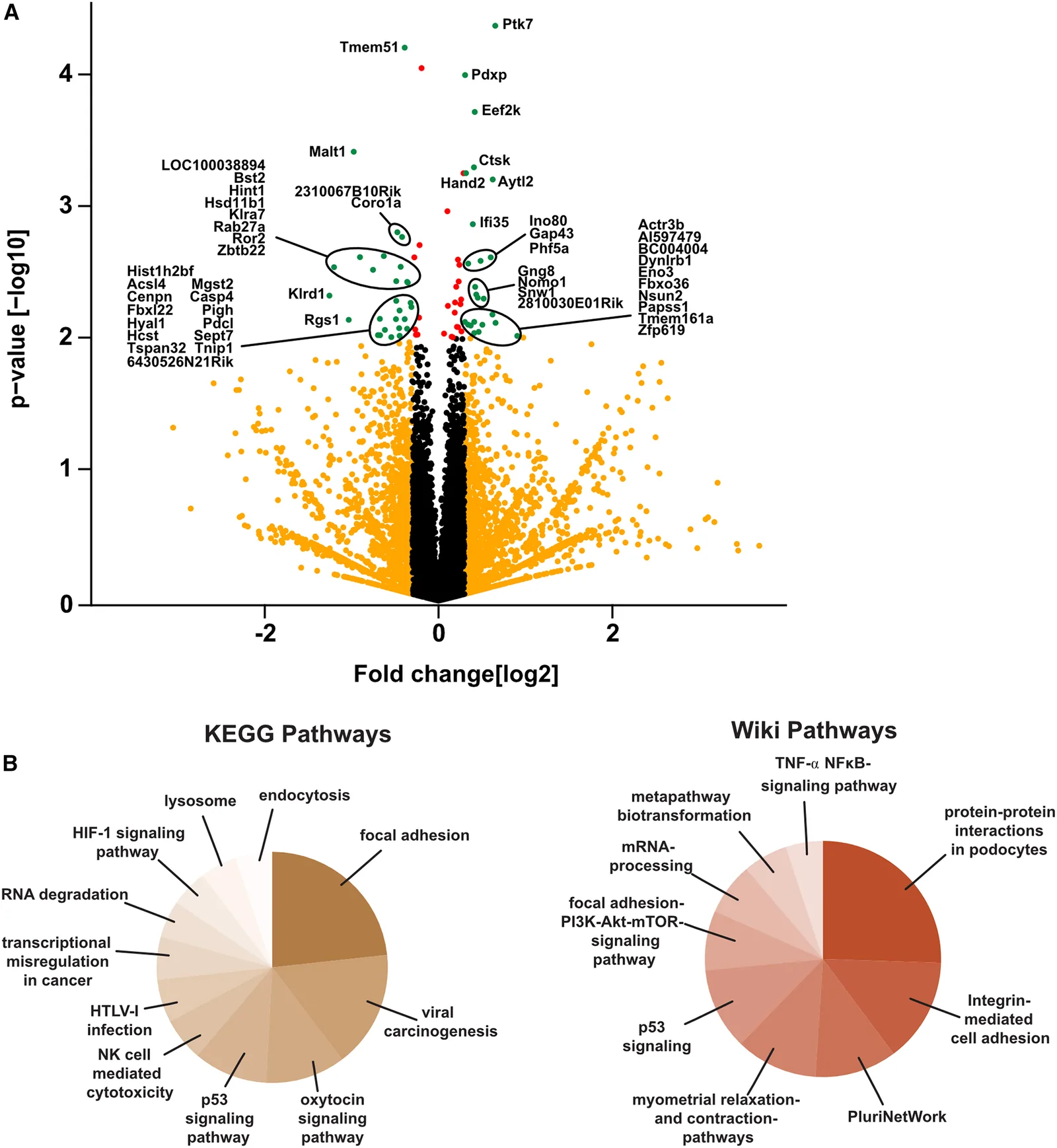
Oxytocin signaling and myometrial contraction-relaxation signaling were impaired in KO mice (A) Transcriptomic analysis of titin KO vs. CON uterus at E3.5. Genes with >0.3 fold-change are depicted in orange. Significantly regulated Genes (*p* < 0.01) are indicated in red. The most regulated genes (*fold change* > 0.3 and *p* < 0.01) are depicted in green, and gene names are provided. (B) Pathway analysis of differentially transcribed genes. Among the enriched terms are oxytocin- and myometrial relaxation and contraction signaling. *n* = 3 per group.

Among the metabolic enzymes affected were Enolase (*Eno3*) and *Microsomal glutathione S-transferase 2* (*Mgst2*) as well as *Regulator of G-protein signaling 1* (*Rgs1*)—the latter linked to the arachidonic acid pathway. Together these findings suggest a broader effect on uterine physiology.

### Increased uterus contractility and response to oxytocin in smooth muscle titin kinase region knockout

Based on the deregulation of genes linked to oxytocin signaling and myometrial relaxation and contraction pathways, we investigated the mechanical properties of titin-deficient uterus using *ex vivo* force measurements on uterus rings. After equilibration for 60 min in physiological saline solution (PSS) including Ca^2+^, Mg^2+^, and glucose, we started treatment with increasing concentrations of oxytocin and recorded the developed forces (Figures 4A and 4B). Maximal contraction was increased in the KO uterus (Figures 4C and 4D). The area under the curve, as a measure of force development, was increased significantly at oxytocin concentrations starting at 0.1 nM in the KO (Figure 4E). Prostaglandin E2 and I2 pre-treatment for 25 min further amplified the oxytocin response (Figures S4A–S4E). Unlike the active properties of smooth muscle, passive properties were unchanged, as resting wall tension was not significantly different between genotypes (Figure 4F). This is consistent with the low expression levels of titin resulting in a minor contribution to the elastic properties.

**Figure 4:**
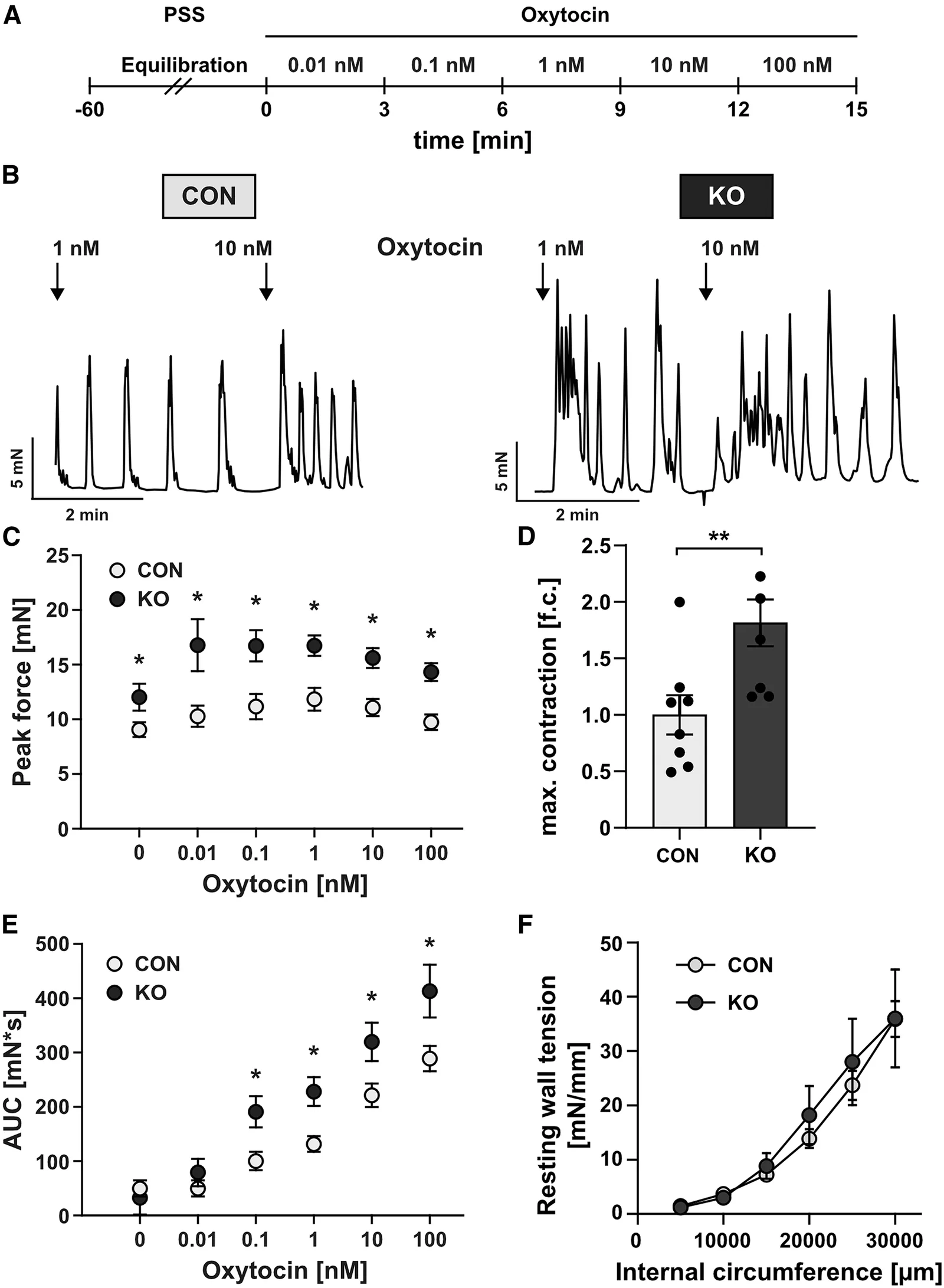
Increased contractile response to oxytocin of titin-deficient uterus rings (A) Experimental design with equilibration time and treatment with increasing oxytocin concentrations in PSS - physiological saline solution. (B) Original traces of isometric forces from Uterus rings of control (CON)- and titin knockout (KO)-animals after stimulation with oxytocin. (C) Peak forces generated by uterus rings from KO animals were stronger as compared to control mice (*n* = 8). (D) Maximal contraction rates were increased >1.8-fold. (E) The area under the curve (AUC) was increased from 0.1 nM oxytocin. *n* = 3 per group with 21–22 rings total; (F) Resting wall tension was not significantly different between genotypes. *n* = 11 per group; repeated measure two-way ANOVA followed by Bonferroni’s post hoc-test was performed to test differences between CON and KO groups. ∗*p* < 0.05, ∗∗*p* < 0.01. Data are presented as mean ± SEM.

To confirm the role of titin in uterus contraction and to dissect the contribution of the longitudinal and circular muscle layers of the uterus to the implantation defect, we replicated our findings in 2D uterus specimen by tracking wall movement under baseline conditions and after oxytocin treatment (Figure 5). Here we tracked motion of the uterus wall in longitudinal and lateral directions, with the latter reflecting the contribution of the circular muscle layer. In both longitudinal and lateral directions, motility became more spatially confined under oxytocin in titin-deficient uteri (Figure 5A), with fewer detected peaks (Figure 5B) despite similar amplitudes (Figure 5C). Thus, the assay indicates reduced bulk tissue movement frequency and spatially restricted wall motion, rather than loss of contractile capacity. Because DiI tracking reports movement of labeled tissue regions rather than single-cell activity, these data do not directly quantify propagation velocity or cellular synchrony. The altered activity in the knockout is an effect directly relevant to embryo crowding.

**Figure 5:**
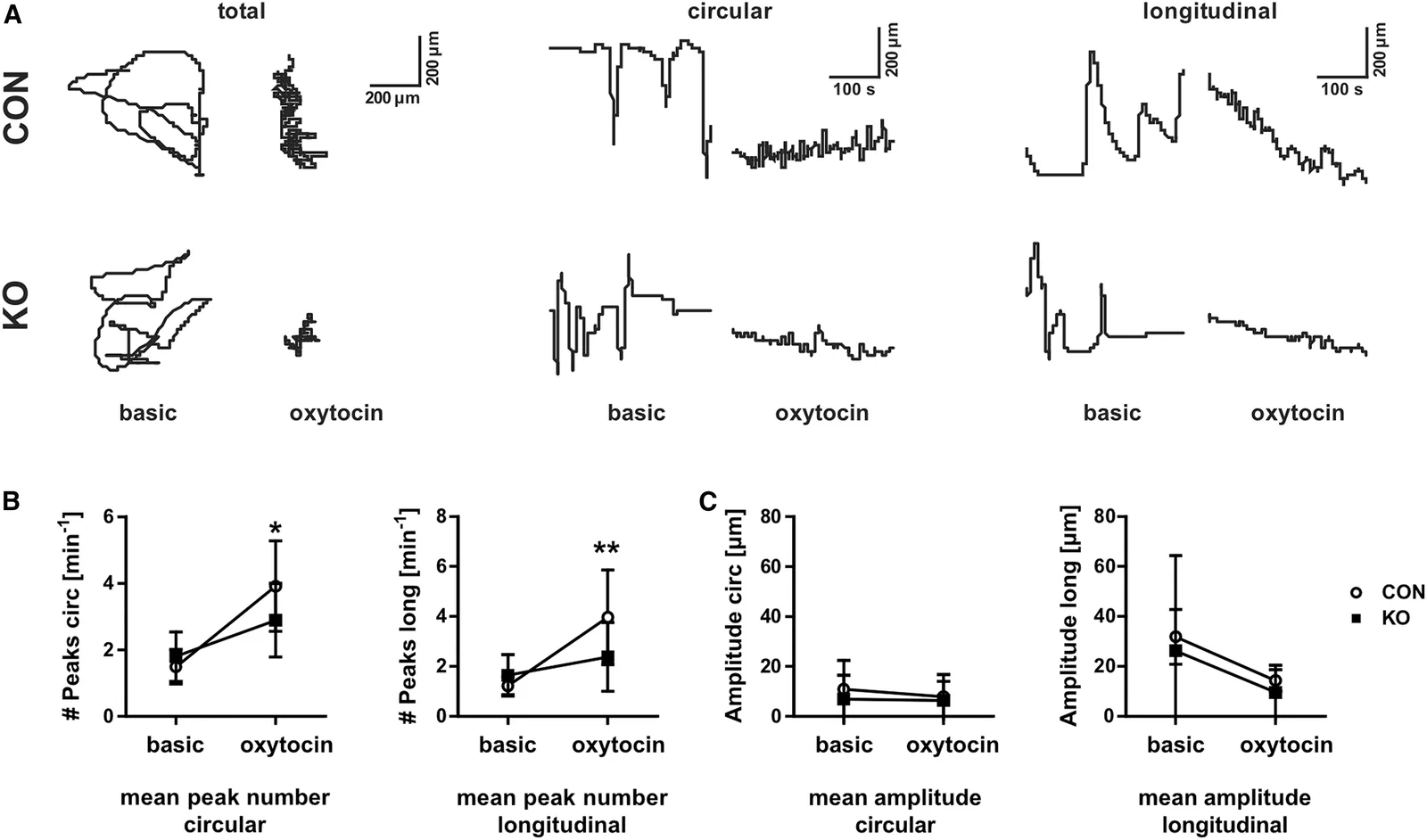
Reduced oxytocin response in titin-deficient uterine wall (A) Tracking the uterus wall movement of control CON and KO uterus in 2D (total movement, left), along circular fibers (middle), and along the longitudinal fibers (right) under control conditions (basic) and after oxytocin treatment (100 nM). (B) Quantification of peak frequency with reduced oxytocin response in the knockout of circular and longitudinal fibers. (C) Similar motility amplitudes in response to oxytocin in CON and KO. Two-way ANOVA followed by Bonferroni’s post hoc test was performed to test differences between CON and KO groups. ∗*p* < 0.05, ∗∗*p* < 0.01. Data are presented as mean ± SEM. Raw data in Figure S5.

### Layer-resolved proteomics identifies myometrial proteostasis, redox, ion-handling, and matrix-remodeling changes after titin M-band deletion

To determine how loss of titin’s M-band kinase region affects associated signaling proteins (Figure 6A), we performed western blotting of uterine tissue and found reduced Nbr1 and increased p62/SQSTM1 protein levels, whereas MuRF1 protein levels were unchanged (Figures 6B and 6C). The combination of NBR1 loss with p62 accumulation is consistent with decoupling of titin-anchored selective autophagy from effective cargo clearance, rather than a uniform up- or down-regulation of all autophagy components. The divergent regulation of p62 and Nbr1 has previously been reported in HeLa cells under toxic stress.^26^

**Figure 6:**
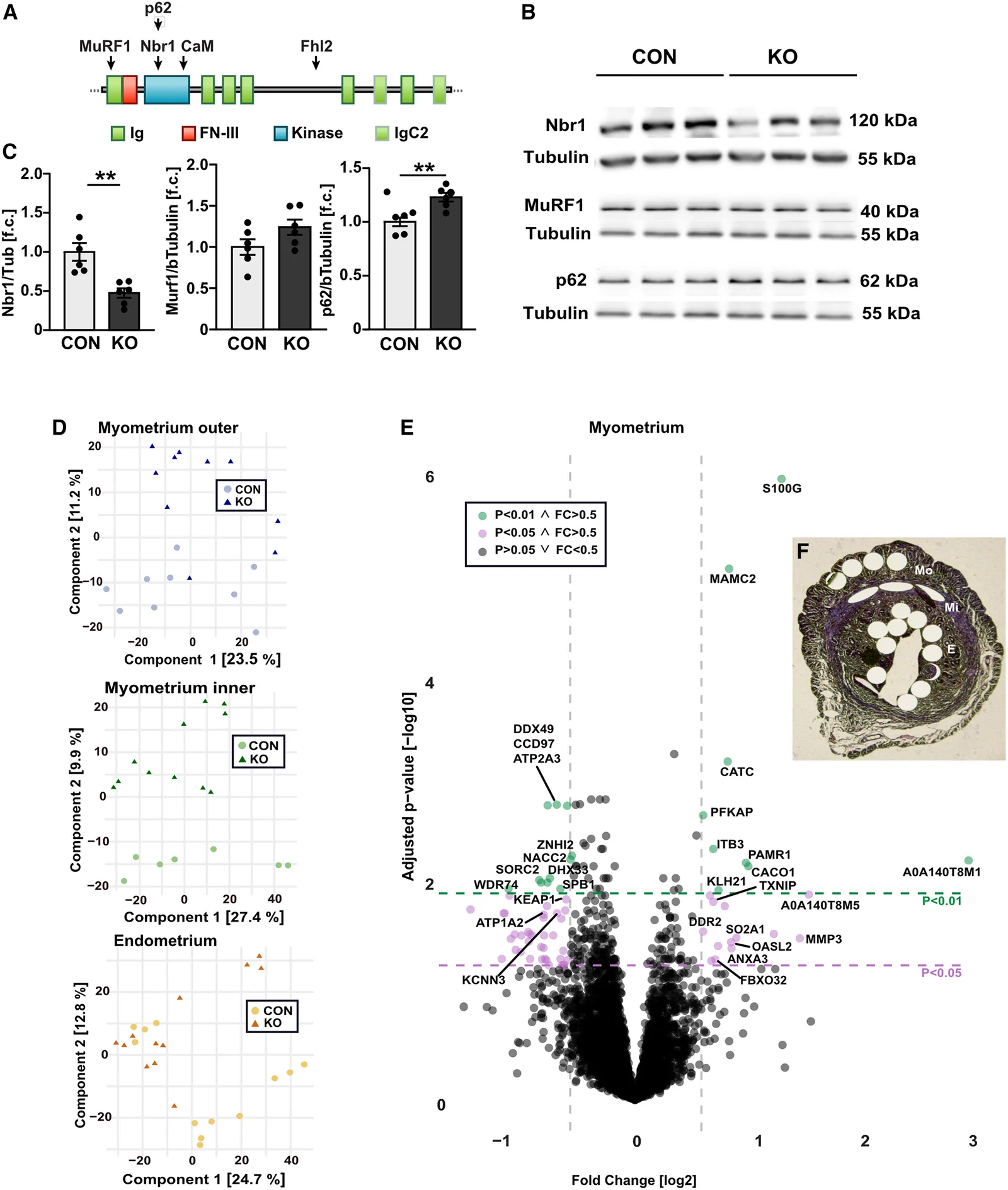
Titin binding proteins were deregulated in the titin M-band-deficient uterus (A) Titin kinase region with binding proteins, immunoglobulin (Ig) and fibronectin (FN) domains. (B) Western blot of titin M-band binding proteins. (C) Quantification of western blot with significant downregulation of Nbr1, unchanged MuRF1 and upregulated p62/SQSTM1, *n* = 6 per group; Student’s *t* test; ∗*p* < 0.05, ∗∗*p* < 0.01. Data are presented as mean ± SEM. (D) Laser-capture microdissection of outer myometrium (Mo), inner myometrium (Mi), and Endometrium (E) followed by proteomics analysis. Myometrium samples show separation between genotypes. (E) Laser microdissected myometrium samples with proteomics analysis of titin KO vs. CON uterus at E3.5. The most regulated proteins (fold change >0.5 and *p* < 0.05) are depicted in green, and protein names are provided. (F) Overview of dissected part of uterus for laser microdissection.

Laser-microdissection-based spatial proteomics revealed that genotype-dependent expression changes were most evident in the myometrium, whereas endometrial samples did not clearly segregate by genotype in principal component analysis (Figure 6D). In myometrium, titin M-band deletion was associated with changes in proteins involved in proteostasis, redox regulation, ion handling, and matrix signaling (Figure 6E). CACO1 was increased, TXNIP was elevated, and KEAP1 was reduced, consistent with altered autophagy-associated and redox-regulatory pathways. FBXO32/Atrogin-1 was also increased, suggesting engagement of ubiquitin-proteasome remodeling. Among excitability-associated proteins, KCNN3/SK3, ATP1A2/Na^+^/K^+^-ATPase α2, and ATP2A3/SERCA3 were reduced, while S100G was increased, consistent with altered Ca^2+^ handling. The prostaglandin transporter SLCO2A1 was increased, suggesting altered prostaglandin handling. At the extracellular interface, MMP3, DDR2, and integrin β3 (ITGB3) were increased. PFKAP was also increased. Pathway enrichment of the proteomics dataset identified focal adhesion, cytoskeleton in muscle cells, spliceosome, integrin-mediated cell adhesion, glycolysis/gluconeogenesis, and oxidative stress/redox pathways (Tables S6 and S7). Together, these data show that titin M-band deletion is associated with myometrial changes in matrix/adhesion proteins, metabolic enzymes, redox-associated proteins, and ion-handling regulators.

Together, the functional and proteomic data suggest that titin M-band deletion does not simply reduce contractile capacity. Instead, the KO uterus remains force-generating but shows altered excitability, relaxation-associated signaling, and tissue coupling. This provides a plausible explanation for increased oxytocin-evoked force together with reduced bulk wall-motion peak frequency and impaired implantation-site distribution.

### Titin M-band loss disrupts myometrial mechanotransduction without reducing contractile protein levels

Western blot validation of whole uterus confirmed significant reductions in smooth muscle α-actin (ACTA2), smooth muscle myosin heavy chain (MYH11), myosin light chain (MLC2), myosin light-chain kinase (MLCK), and telokin in knockout uteri (Figure S6). In the laser micro-dissected myometrium, these proteins were unchanged, suggesting that intrinsic force generation within myometrial smooth muscle was not impaired (Figure 6E).

Functional assays supported these findings. *Ex vivo* ring myography demonstrated increased peak force (Figure 4C) and area-under-the-curve responses to oxytocin in knockout uteri (Figure 4E), while wall movements under baseline conditions were unchanged (Figure 5), indicating no effect on baseline tissue stiffness. Stimulated wall-motion tracking by oxytocin showed reduced numbers of peaks and more spatially confined displacements in KO uterine strips, reflecting impaired coordination of contractile waves (Figure 5B). *In vivo*, knockout females had fewer implantation sites, with evidence of crowding and mislocalization (Figure 2A). At term, placental weights were increased, consistent with compensatory adaptation to suboptimal implantation spacing (Figure 2F).

## Discussion

In this study, we used a loss-of-function approach to document the existence of smooth muscle titin and supported these findings with a refined analysis of titin expression with enhanced vertical agarose gel electrophoresis (VAGE) and mass spectrometry to identify full-length titin protein in the uterus. In the smooth muscle titin knockout, we find embryo implantation defects with fewer implantation sites, mislocalization and crowding.

In the uterus, smooth muscle contractions support the movement of embryos along the oviduct prior to implantation, as blastocysts distribute equally along the mouse uterine horns.^27^^,^^28^^,^^29^^,^^30^ Myometrial contractions are elevated during the preimplantation period and relaxin, an inhibitor of myometrial activity, disrupts the blastocyst distribution in rats.^31^^,^^32^^,^^33^ Multiple pathways converge on myometrial contraction—including adrenergic signaling,^34^ prostaglandin metabolism,^35^^,^^36^^,^^37^ and cannabinoid signaling,^38^ but we are only beginning to understand how these signals are integrated downstream at the level of the myofilament, which is quite different between striated and smooth muscle.

In uterine smooth muscle, levels of full-length titin are more than two orders of magnitude lower than in striated muscle (Figure 1). The resulting minor contribution of titin to myometrial stiffness would suggest a mechanism where titin is not the determinant but rather the sensor of the biomechanical properties. Indeed, the titin kinase domain has been described as mechanically activated, and despite the controversy around its catalytic activity, its role in mechanotransduction is generally accepted.^39^^,^^40^^,^^41^ Here, forces sufficiently strong to separate the regulatory and catalytic domains expose the titin kinase region as a hub for a signaling complex that links to proteostasis and hypertrophy signaling in striated muscle and includes an Nbr1, p62, and Murf1 adaptor module that can engage MuRF-family ubiquitin ligases.^9^^,^^39^ In the uterus, deletion of this domain selectively collapses the autophagy node, lowering NBR1 while increasing p62, and triggers a compensatory upshift of CACO1 together with TXNIP upregulation and KEAP1 reduction, indicating activation of the NRF2-dependent redox pathway. The proteasomal marker FBXO32 increased, further confirming stress-induced proteostasis remodeling.

Downstream, excitability-associated proteins were altered, with S100G strongly upregulated and the Ca^2+^-handling pumps ATP1A2 and ATP2A3 reduced, indicating impaired Ca^2+^ re-uptake and enhanced intracellular Ca^2+^ sensitivity. KCNN3 (alias SK3) downregulation removes a hyperpolarizing brake, while SO2A1 upregulation reduces prostaglandin-mediated relaxation. Together, these changes yield a hyperexcitable, Ca^2+^-sensitized myometrium.

Matrix-adhesion signaling was simultaneously reinforced, as MMP3, DDR2, and integrin β3 were increased, supporting ECM turnover and altered viscoelastic coupling. This remodeling likely amplifies local traction forces but disrupts coordinated wave propagation between muscle layers.

Metabolic compensation was evident through increased glycolytic enzyme PFKAP, consistent with elevated energetic demand in the hypercontractile tissue. Importantly, core contractile proteins remained unchanged in the isolated myometrium, indicating that functional hypercontractility arises from signaling and coupling reprogramming rather than from increased myofilament content.

Collectively, these results redefine the uterine titin M-band as a signaling integrator rather than a structural determinant. Its loss is associated with disruption of an NBR1/p62-associated proteostasis module, but the present study does not directly demonstrate physical interaction between uterine titin and NBR1/p62 or measure autophagy flux. Future co-immunoprecipitation, proximity-labeling, NBR1/p62 localization, and LC3/p62 turnover experiments are needed to define the direct molecular coupling.

Functionally, titin M-band deletion produced an apparent paradox: KO uteri generated increased oxytocin-evoked peak force and area under the curve, yet bulk wall-motion tracking revealed fewer movement peaks and more spatially confined motion. Thus, the KO uterus does not appear to have reduced contractile capacity. Rather, it remains strongly force-generating but displays altered active contractile regulation and reduced bulk wall-motion coordination. Importantly, the DiI tracking assay follows movement of labeled tissue regions and therefore reports tissue-level motion rather than single-cell propagation velocity or cellular synchrony. We therefore describe this phenotype as reduced bulk wall-motion peak frequency and spatially restricted motion, rather than directly measured propagation velocity or synchrony.

Our findings have direct translational implications: In humans, excessive or poorly coordinated uterine contractions around embryo transfer are associated with reduced *in vitro* fertilization (IVF) success.^42^ We propose that intrinsic defects in mechanotransduction, including variants in *TTN* (titin) gene or its interactors (NBR1, p62, Murf1), may contribute to unexplained infertility. Biomarkers such as autophagy adaptor levels or autophagy/ECM signatures could serve as indicators of risk for implantation failure. Moreover, pharmacological modulation of these pathways—cGMP/PKG activators to restore telokin signaling, agents that stabilize calcium buffering (S100G modulators), or agents that restore redox and autophagy balance—may offer novel strategies to optimize peri-implantation uterine motility.

In clinical genetics, *TTN* truncating variants (TTNtv) are common causes of dilated and peripartum cardiomyopathies; multiple cohorts show TTNtv enrichment in women with peripartum cardiomyopathy, underscoring pregnancy as a stress test for titin function in muscle.^43^ However, we found no direct human evidence that *TTN* variants cause implantation failure or uterine hypercontractility—this is an open gap that our model motivates to test in reproductive cohorts. Large registries (cardiomyopathy genetics and assisted reproductive technology (ART)) could enable fertility/implantation phenotyping in *TTN* carriers, including uterine motility readouts and perinatal outcomes.

### Limitations of the study

The difference between whole-uterus western blotting and laser-captured myometrial proteomics likely reflects tissue composition. Whole uterus lysates contain myometrium, endometrium, perimetrium, vasculature, stroma, and immune cells, whereas the LCM proteomics specifically enriches the myometrial layer. Therefore, reduced ACTA2, MYH11, MLC2, MLCK, and telokin in whole uterus may reflect changes in the relative contribution or state of non-myometrial compartments, regional heterogeneity, or tissue-level remodeling, rather than a uniform loss of these proteins within smooth muscle cells. This interpretation is consistent with the preserved abundance of these proteins in laser-captured myometrium and with the retained, indeed increased, contractile response in organ bath experiments.

Translation of our findings to human fertility requires caution. Mice have a bicornuate uterus with multiple implantation sites along two uterine horns, whereas humans have a single uterine cavity with different implantation dynamics. Future studies using human myometrial tissue, primary uterine smooth muscle cells, or organoid/co-culture systems will be needed to determine whether titin-dependent mechanotransduction contributes to human implantation failure or uterine contractility disorders.

## Resource availability

### Lead contact

Requests for further information and resources should be directed to the lead contact, gotthardt@mdc-berlin.de.

### Materials availability

The study did not generate any new unique materials.

### Data and code availability

• The mass spectrometry proteomics data generated in this study have been deposited in the ProteomeXchange Consortium via the PRIDE partner repository and are publicly available with the dataset identifier PXD083488. The Illumina microarray data generated in this study have been deposited in the ArrayExpress database at EMBL-EBI under the accession number E-MTAB-17587.
• This paper does not report original code.
• Any additional information required in this paper is available from the lead contact upon request.

## Acknowledgments

This work was funded by the German Research Foundation (DFG to M.Got. and M.Gol.). M.K. and F.C. acknowledge funding support by the Federal Ministry of Education and Research (BMBF), as part of the National Research Initiatives for Mass Spectrometry in Systems Medicine, under grant agreement no 161L0222. We are grateful to Janine Fröhlich, Carmen Judis, Isyatul Azizah, Ning Wang and Beate Goldbrich-Hannig for expert technical assistance.

## Author contributions

V.J.S., N.B., C.F., R.J., L.S.A., M.B., J.E.F. performed the research and generated display items, V.J.S., C.F., and N.B. designed the animal experiments, J.E.F. and M.B. performed the life time imaging, M.K., F.C., L.S.A., and P.M. generated and analyzed the proteomics dataset, R.J. and M.Gol. designed, performed, and analyzed the biomechanics experiments, M.Got. conceived the project, analyzed data, generated display items, and wrote the manuscript with input from all authors.

## Declaration of interests

The authors declare no competing interests.

## Declaration of generative AI and AI-assisted technologies in the manuscript preparation process

ChatGPT was used for language editing, document organization, and formatting support. All analyses, scientific interpretations, references, and final wording were reviewed and approved by the authors, who remain fully responsible for the content.

## STAR★Methods

### Key resources table

**Table:** 

REAGENT or RESOURCE	SOURCE	IDENTIFIER
**Antibodies**		

ACTA22	Sigma-Aldrich	A2547
Beta-Tubulin	Cell Signaling	2146
Mlc2	Cell Signaling	8505
MuRF1	Abcam	Ab77577
Nbr1	Cell Signaling	9891
p62	Abcam	Ab53219
Smmhc	Abcam	Ab53219
Mylk	Abcam	Ab76092

**Experimental models: Organisms/strains**		

Mex1-2 KO mice	Own	Mex1-2 KO
GtROSA26 reporter mice	The Jackson Laboratory	Catalog No. 003309
SMMHCcre transgenic mice	Robert Feil	SMMHCcre
titin-GFP knock-in	own	titin-GFP
TiEx28dsRed	own	TiEx28dsRed

**Oligonucleotides**		

Table S8	Table S8	Table S8

**Deposited data**		

Illumina microarray gene expression data	ArrayExpress	E-MTAB-17587
Targeted mass spectrometry and spatial proteomics	PRIDE	PXD083488

**Software and algorithms**		

GraphPad Prism 5.0 and 8.0	Graphpad software	RRID: SCR_002798
LabChart 8	ADInstruments	Version 8
SymPhoTime 64	PicoQuant GmbH	Version 2.11
Sequence Detection System	Applied Biosystems	Version 2.3
Laser Microdissection software	Leica Microsystems	Version 8.3
Leica LMD7 system	Leica Microsystems	LMD7chart
Chart 5	ADInstruments Ltd, Oxford,UK	Chart5
Fiji (ImageJ)	Fiji/ImageJ	RRID: SCR_002285
ClueGO (v2.5.10)	Cytoscape app	RRID: SCR_005748
R	The R foundation	Version 4.3.3

### Experiment model and study participant details

#### Animals

The MEx1-2 KO mice with *loxP* sites flanking titin's M-band exons 1 and 2 have been described previously.^21^ To achieve smooth muscle-specific recombination, floxed mice were crossed with SMMHC-Cre mice expressing Cre recombinase under the control of the smooth muscle myosin heavy chain promoter.^22^ Successful recombination and deletion efficiency were validated by genotyping PCR and analysis of titin expression in smooth muscle tissue (Figure S1). All experiments involving animals were carried out following institutional and National Institutes of Health guidelines and follow the Directive 2010/63/EU of the European Parliament on the protection of animals used for scientific purposes and have been approved by the local German authorities (LaGeSo Berlin, Germany), approval numbers G0038/18 and X9011/19. Mice were maintained on a 129/S6 background and housed under specific pathogen-free conditions in individually ventilated cages with free access to food and water under a 12 h light/dark cycle at 22 ± 2°C and 55 ± 10% humidity. Female mice older than 100 days were used for timed matings, and estrous cycle stages were monitored. Only female mice were included because the present study specifically investigates uterine and reproductive physiology; therefore, assessment of sex-dependent differences between male and female animals was not applicable to the primary study question.

GtROSA26 reporter mice were from The Jackson Laboratory (Catalog No. 003309). SMMHCcre mice were a generous gift from Robert Feil. SMcGtROSA26 mice were generated by crossing SMMHCcre mice with GtROSA26 reporter mice.

### Method details

#### Estrous cycle staging

To collect non-pregnant uteri in the estrous stage of the estrous cycle and to determine the estrous cycle length, it was necessary to monitor changes in vaginal epithelial cells at 24 h intervals by vaginal lavage.^44^ Therefore, the vagina of 90 days old wild-type, heterozygous, and homozygous TiEx28dsRed,^45^ double heterozygous and heterozygous SMcGtROSA26 mice, SMMHCcre mice, and control, heterozygous, and knockout SMcTiMEx1-2 female mice was rinsed with ddH_2_O to extract vaginal cells and the fluid was placed onto a microscope slide. After evaporation of the water, the sample was examined under the inverted light microscope Olympus CK40 applying the guidelines of Allen and Snell.^46^ At least, two continuous cycles were observed before sacrificing the mice.

#### Timed matings and analysis of implantation

For timed pregnancies, 90-day-old females of the indicated genotypes were mated with males selected to preserve the required experimental genotype while avoiding female germline recombination associated with SMMHC-Cre. Control females were mated with males carrying the appropriate floxed titin allele, whereas heterozygous and knockout females were mated with control males as required for the breeding strategy. Mating was set up at 6 p.m. and the morning of detecting a vaginal plug was designated as E0.5. To count blastocysts on the stereomicroscope Leica MZ7.5, both uterine horns were flushed twice with PBS.

Embryo implantation sites at E4.5 and E5.5 were stained by intravenous injection with 150 μL Evans Blue (1% in PBS) solution.^47^ 5 min after injection uterus and heart were dissected. Their weight was determined and pictures of the uteri were taken using an Olympus μ 720 SW camera. Afterward uterus horns were investigated for the presence of blastocysts.

The GtROSA26 reporter mice were used only to validate SMMHC-Cre recombination in uterine smooth muscle, and titin-GFP knock-in mice were used only for localization/lifetime imaging of titin in uterine smooth muscle cells.

#### Myography

Contractile properties of isolated uteri in estrous phase were analyzed from female mice (20–30 g, 15–25 weeks, estrous cycle). Mice were sacrificed and the uterus was removed, quickly transferred to cold (4°C) oxygenated (95% O_2_/5% CO_2_) physiological salt solution (PSS), and dissected into 3 mm rings, respectively. After 1 h of equilibration, isometric contractile force of uterus rings was measured using a Schuler Tissue Bath System (Hugo Sachs, Freiburg, Germany). The composition of PSS in the bath chamber was (in mmol/l) 119 NaCl, 4.7 KCl, 1.2 KH_2_PO_4_, 25 NaHCO_3_, 1.2 MgSO_4_, 11.1 glucose, and 1.6 CaCl_2_. Passive properties were measured using a Multi Wire Myograph System (Danish Myo Technology A/S, Hinnerup, Denmark).^48^ The software Chart5 (ADInstruments Ltd Germany, Oxford, UK) was used for data acquisition and display. Basal tone was pre-adjusted to 0.5 g and contractile force was continuously monitored. The bath solution volume was 20 mL. In a first set of experiments, isometric contractions in response to increasing concentrations of oxytocin (range, 10^−11^ to 10^−7^ M) were measured. In a second set of experiments, isometric contractions in response to 1 μM prostaglandin E2 (PGE_2_) plus 1 μM prostaglandin I2 (PGI_2_) were measured. Thereafter, oxytocin (range, 10^−11^ to 10^−7^ M) was added to the bath chamber and contractions were recorded. Peak contractions, frequencies of contractions (spikes) and area under the curves (AUC) were calculated using software LabChart 8 (ADInstruments Ltd Germany, Oxford, UK). Spike threshold was 1 mN. AUC were calculated from 2-min periods of recordings for each concentration of oxytocin. Average values were calculated using GraphPad Prism 5 software (GraphPad Software, Inc., La Jolla, CA, USA).

#### DiI tracking

After cervical dislocation of female mice, the uterus was quickly removed and cleaned to dissect two longitudinal strips that were pinned on a sylgard-coated Petri dish (Sylgard 184, MERCK) using minutien pins (0.2 mm, Fine Science Tools). Preparations were analyzed in a buffer containing (in mM): 125 NaCl, 3 KCl, 1.25 NaH_2_PO_4_, 26 NaHCO_3_, 10 glucose, 2 CaCl_2_, 1 MgCl_2_, adjusted to pH 7.3. The strips were labeled with DiI (DiIC18(3), Molecular Probes) for 60 min at 37°C (for orientation compare Figure S5A). Movements of the stripes were visualized on a fluorescence microscope (Examiner A1, Zeiss, excitation at 540 nm, filterset 1, Zeiss) and recorded for 5 min using Live Acquisition (Till Photonics). For data processing and analysis we used Tracker (Douglas Brown, Open Source Physics) and CaSiAn (Calcium Signaling Analyzer).^49^

#### Histology

Heart and skeletal muscle tissue were fixed in 4% paraformaldehyde and embedded in paraffin. Sections were stained with Toluidine Blue (0.05% for 2 min) as described previously.^50^

#### KEGG and reactome pathway enrichment analysis

All analyses were done using the CLUEGO plugin in Cytoscape.^51^^,^^52^ Kappa-score threshold was set to 0.3, min % genes to 0 and min number of genes to 2. *p-values* were Benjamini-Hochberg corrected for multiple testing and denoted *(p-adj.*).

#### Gene expression analysis

Uterus and skeletal muscle samples were washed in PBS, snap frozen in liquid nitrogen, and stored at −80 °C. For RNA extraction we used the RNeasy Mini kit (Qiagen) with DNAse digestion as per the provider’s protocol. RNA was reverse transcribed into cDNA using the ThermoScript RT-PCR System for First-Strand Synthesis Kit with random hexamer primers. To detect low abundant titin mRNA with the primer/probe sets MEx1-2, MEx6 TaqMan was carried out with the RNA UltraSense One-Step Quantitative RT-PCR System in triplicates on a 7900HT Fast Real-Time PCR System (Applied Biosystems). Information on the amplicons is provided in the online supplement (Table S8). Data were collected with the Sequence Detection System 2.3 software (Applied Biosystems) and analyzed using the Comparative CT Method. For this relative quantification, data were normalized to 18S cDNA and displayed as fold change relative to the control. Proteins were extracted with RIPA buffer (50 mM Tris pH 8; 150 mM NaCl; 1% IGEPAL; 0.1% Na DOC; 5 mM EDTA; 0.1% SDS; protease and phosphatase inhibitor complex (Invitrogen)) and separated by SDS-PAGE. Western blot was performed on PVDF membranes. Antibodies are listed in the supplement (Table S7). The secondary HRP conjugated antibody was detected by chemiluminescence staining with ECL (Supersignal West Femto Chemiluminescent Substrate; Pierce Chemical Co.). To separate titin isoforms we used vertical SDS agarose gel electrophoresis (VAGE) as described.^53^ Briefly, tissue samples were homogenized and diluted 1:10 in titin loading buffer. The sample buffer contained 8 M urea, 2 M thiourea, 3% SDS w/v, 75 mM DTT, 0.03% bromophenol blue, and 0.05 M Tris HCl, pH 6.8. After incubation in loading buffer for 4 min at room temperature, samples were incubated for 30 min at room temperature and centrifuged at 13,000 rpm for 5 min. The supernatants were collected and stored at −80 °C until use. Samples were separated on vertical agarose gels under constant-current conditions (6.5 mA) overnight at 4 °C. For each lane, 10 μL sample was loaded unless otherwise indicated. “Overloading” refers to loading 30 μL sample per lane to improve visualization of high-molecular-weight titin bands. Running buffer was freshly prepared before electrophoresis.

#### Illumina microarrays

Tissue was homogenized with a homogenizer in trizol (Invitrogen), extracted with chloroform, precipitated in ethanol, and dissolved in nuclease-free water. After quality control (2100 Bioanalyzer, Agilent) and DNAse digestion, RNA was purified using RNeasy columns (Qiagen) and stored at −80 °C. To reduce ribosomal RNA (rRNA) we used the RiboMinus Kit (Invitrogen). Biotinylated cRNA was prepared using the Illumina® TotalPrep-96 RNA Amplification Kit (Life technologies) according to the manufacturer’s directions starting with ∼100 ng total RNA. Expression profiling was performed using Illumina’s MouseRef-8 v2.0 Expression Bead chip. Chips were quantile normalized in Genome Studio V2011.1 using the Gene Expression Module V1.9.0. Sample gene profile data was used for further analysis. Only genes that were expressed at levels significantly above background were considered (*p-value* < 0.05). Differentially expressed genes were considered to be those with a *p-value* < 0.05.

#### Protein samples preparation for mass spectrometry

Uterine smooth muscle cells were isolated following method as described.^54^ Cells were cultured in T25 flasks until confluent and scraped off in RIPA buffer followed by incubation on ice for 30 min and 3 × 10 Impulses sonication (Hielscher vial tweeter) 70 amplitude, 0.5 s/cycle and centrifugation for 20 min at 4 °C in 13000 rpm.

#### Peptide identification by mass spectrometry

After protein separation using polyacrylamide gel electrophoresis (PAGE), titin bands were cut and gel pieces were subjected to tryptic in-gel digestion^55^ followed by stage-tip peptide desalting. Samples were measured by LC-MS/MS on a Q Exactive Plus mass spectrometer (Thermo) connected to an EASY-nLC system (Thermo). Database search was performed with MaxQuant version 1.6.10.43^56^ adding Oxidation (M) and N-terminal acetylation as variable modifications. An FDR of 0.01 was applied for peptides and proteins and the Andromeda search was performed using a mouse Uniprot database (UP000000589) using the match-between-runs feature. Decoy hits and contaminants were removed. For downstream analysis and plotting, peptide evidence entries were summed across biological replicates for Uterus and Gastrocnemius sample based on the “Experiment” annotation, only peptide evidence identified by MS/MS spectra was included in the quantification.

#### Immunofluorescence of Uteri for lifetime imaging

Mouse uteri were collected, briefly rinsed in cold PBS, and fixed in 4% paraformaldehyde (PFA) for 12–16 h at 4°C. Following fixation, tissues were cryoprotected in 30% sucrose overnight, embedded in Tissue-Tek OCT compound, and frozen using dry ice-cooled isopentane. Cryosections (5–10 μm) were cut using a cryostat and mounted onto Superfrost Plus microscope slides. Sections were air-dried, encircled with a hydrophobic pen, and permeabilized with 0.1% Triton X-100 in PBS (PBST) for 15 min. After blocking with 5% normal serum for 1 h at room temperature, sections were incubated overnight at 4°C with primary antibodies diluted in PBST. The following day, slides were washed and mounted using gold anti-fade mounting medium.

#### Lifetime imaging

FLIM was conducted using a PicoQuant system with a TCSPC MicroTime 200 module on an inverted Olympus IX83 microscope with a 60× 1.4-NA water-immersion objective. Pulsed Interleaved Excitation at 479 nm and 680 nm (26.76 MHz) enabled fluorophore separation, with a triple-line beamsplitter directing signals to SPAD detectors. Images (300 × 300 px, 10 μs dwell) were acquired in lifetime mode, processed using the SymPhoTime 64 software, and intensity-based images were extracted in Fiji. Fluorescence lifetimes were fitted with a 3-component n-Exponential Reconvolution fit, incorporating the instrument response function. Data were thresholded, smoothed, and exported as bitmaps, with histograms and fitting properties compiled in Prism GraphPad for visualization.

#### Laser microdissection

The Leica LMD 7 system and Laser Microdissection software (version 8.3) was used for the collection of tissue contours. Tissue was cut with a 20× objective (HC PL FL L 20×/0.40 CORR) in brightfield mode. We used the following laser settings: power 57, aperture 1, speed 13, middle pulse count 2, final pulse 1, head current 57% (depending on tissue type and section thickness), pulse frequency 2055 and offset 110. Tissue contours were collected manually into a low-binding 384-well plate (Eppendorf 0030129547) configured over the ‘universal holder’ function with one empty well between samples.

For mouse uterus tissue samples (5 μm-thick sections cut with a microtome), Endometrium, inner and outer Myometrium regions of 30000 μm2–45000 μm2 were collected. Efficient collection was ensured by well inspection.

#### Proteomics sample preparation

Laser microdissected samples were lysed, reduced and alkylated in 2 μL lysis buffer (0.1% DDM, 5 mM TCEP, 20 mM CAA in 100 mM TEAB pH 8.5 in water). Plates were closed with a PCR ComfortLid (Hamilton) and incubated for 1h at 95°C in a thermal cycler. Samples were cooled down and proteins were sequentially digested. First, 1 μL Lys-C (prediluted in water to 2 ng/μL) was added and digested for at least 2h at 37°C in a thermal cycler (lid temperature: 50°C). Following, 1 μL trypsin (prediluted in water to 2 ng/μL) was added and incubated in the thermal cycler at 37°C overnight. The reaction was quenched with 1 μL TFA (1% final concentration) and vacuum dried (20 min at 60°C) before peptide-cleanup.

#### Peptide cleanup with C18-tips

Peptide cleanup was performed with Evotips (EV2011, Evosep, Odense, Denmark) as recommended by the manufacturer with all centrifugation steps being performed for 1 min at 800 g. Briefly, Evotips were rinsed with 20 μl Solvent B (0.1% FA in ACN). The tips were conditioned by soaking in Isopropanol for approx. 10 s and equilibrated by adding 20 μL Solvent A (0.1% FA in LC-MS grade water). The peptide samples in the 384 well plate were resuspended in Buffer A, loaded to the Evotip and once washed. Cleaned Peptides were eluted with 20 μL Buffer into a 96-well plate and vacuum dried (15 min at 60°C). The plate was stored at −20°C until LC-MS measurement. Before analysis, 4.2 μL of MS loading buffer (3% ACN, 0.1% FA in LC-MS water) were added, briefly vortexed and centrifuged. The plate was closed with a silicon mat, 4 μL sample were injected for MS analysis.

#### Liquid chromatography–mass spectrometry (LC – MS) analysis

For LC-MS analysis, we used a setup of an EASYnLC-1200 system (Thermo Fisher Scientific) connected to a trapped ion mobility spectrometry quadrupole time-of-flight mass spectrometer (timsTOF Ultra2, Bruker Daltonik) with a nano-electrospray ion source (CaptiveSpray, Bruker Daltonik). The autosampler was configured to pick samples from 96-well plates. Peptides were separated on a 20-cm home-packed HPLC column (75-µm inner diameter packed with 1.9-µm ReproSil-Pur C18-AQ silica beads, Dr. Maisch), using a linear 44 min gradient at a flow rate 250 nL/min. The gradient consisted of an increase from 5% to 30% buffer B over 29 min, followed by a ramp to 60% buffer B over 3 min. This was succeeded by a 5 min wash at 90% buffer B and a final re-equilibrating step of 5 min at 50% buffer B. The total gradient length was 44 min. Column temperature was kept consistent at 40 °C using a column oven. For dia-PASEF analysis, we used a dia-PASEF method with 8 dia-PASEF scans separated into 3 ion mobility windows per scan covering a 400–1000 m/z range by 25 Th windows and an ion mobility range from 0.64 to 1.37 Vs. cm-2. The mass spectrometer was operated in high sensitivity mode, with an accumulation and ramp time at 100 ms, capillary voltage set to 1750V and the collision energy as a linear ramp from 20 eV at 1/K0 = 0.6 Vs. cm-2 to 59 eV at 1/K0 = 1.6 Vs. cm-2. The collision energy was ramped linearly as a function of ion mobility from 59 eV at 1/K0 = 1.6 V s cm-2 to 20 eV at 1/K0 = 0.6 V s cm-2.

#### Proteomics raw data analysis

Proteomics raw files were processed using DIA-NN (version 2.0 Academia) with a predicted mouse library (Uniprot release 2023). DIA-NN was operated in the default mode with minor adjustments. Briefly, MS1 and MS2 accuracies were set to 15.0 and MBR enabled. Missed cleavages were set to 2 maximum number variable modifications to one with N-terminal M excision, C carbamidomethylation and oxidation(M) selected.

### Quantification and statistical analysis

Proteomics data analysis was performed with Perseus (version 2.1.2.0) and within the R environment (https://www.r-project.org/, version 4.3.3) with the following packages: dplyr (v1.1.4), tidyr (v1.3.1), ggplot2 (v3.5.1), data.table (v1.17.0), ggrepel (v0.9.6). Based on Principal component analysis, one outer myometrium sample was removed. Here, not only inner and outer myometrium but also adjacent endometrium was isolated by laser microdissection. For differential expression analysis (*t* test), data were filtered by tissue layer and only proteins with 70% non-missing data in at least one group were kept. Missing values were imputed based on a normal distribution (width = 0.3, downshift = 1.8) before statistical testing. *t* test parameters were set to permutation-based FDR = 0.01, S0 = 0.1, numbers of randomization = 250. GraphPad prism (version 8.0, GraphPad Software, USA) software was used for statistical analysis. Results are expressed as means ± SEM. Western blot band intensities were quantified by densitometric analysis using Fiji/ImageJ software. Statistical tests are indicated in each figure legend. All tests were two-sided. A *p* value <0.05 was considered as statistically significant (∗), (∗*p* < 0.05, ∗∗*p* < 0.01, ∗∗∗*p* < 0.001, ∗∗∗∗*p* < 0.0001).

## Supplemental information

Document S1. Figures S1–S6 and Tables S2–S9, and Data S1

Table S1. Peptide information related to uterine titin
