# Supplementary material for: The smooth muscle titin filament system regulates uterus contractility and implantation
Source: iScience. 2026 Oct 14;29(11):117888. doi: 10.1016/j.isci.2026.117888 (PMC13694822; doi:10.1016/j.isci.2026.117888)

iScience, Volume *29*

Supplemental information

The smooth muscle titin filament system regulates uterus contractility and implantation

V Juhi Sur, Claudia Fink, Nora Bergmann, Rene Jüttner, Melissa Klingeberg, Fabian Coscia, Lorena Suarez-Artiles, Philipp Mertins, Johanna Elisabeth Fritsch, Melissa Birol, Maik Gollasch, and Michael Gotthardt

Supplemental figures

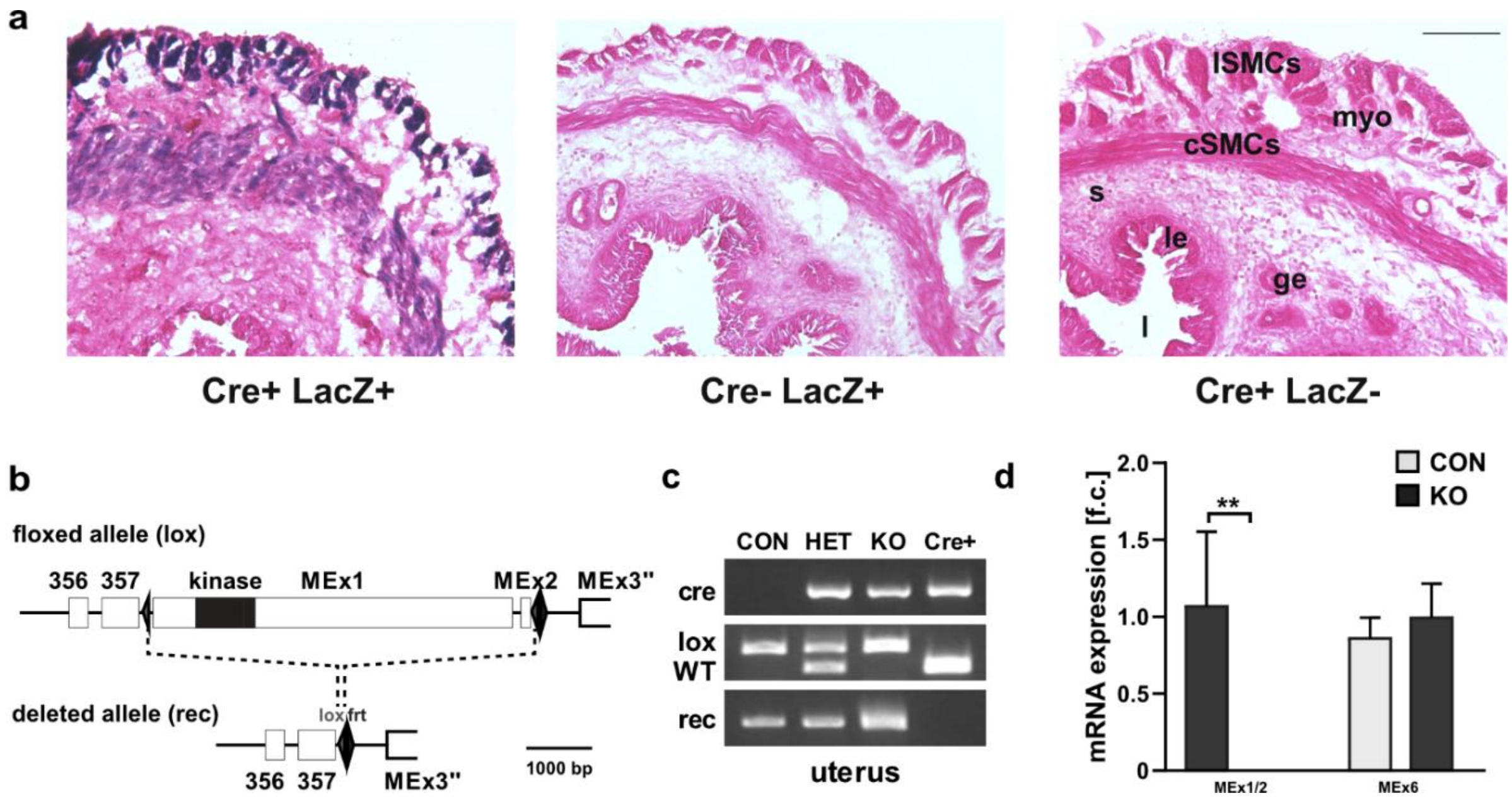

Figure S1: Expression of full-length titin mRNA in the myometrium and depletion of M-band Exon 1 and 2 in the uterine knockout. **a** Activity of the SMMHC cre-recombinase in uterine smooth muscle. LacZ staining (blue) of inner circular and outer longitudinal smooth muscle cells in the uterus of animals that contain the lacZ reporter gene as well as the cre-recombinase transgene (Cre+ lacZ+). Negative controls were Cre- lacZ+ and Cre+ lacZ- only with eosin counterstaining (red). cSMCs: circular smooth muscle cells; ge: glandular epithelium; l: lumen; le: luminal epithelium; lSMCs: longitudinal smooth muscle cells; myo: myometrium; s: stroma; size bar: 100 µm. **b** Expression of the cre recombinase under control of the SM-MHC promoter removes M-band exon 1 and 2 (MEx1-2) including the titin kinase region from the myometrium. **c** PCR-based genotyping of uterine tissue from floxed control, heterozygous, homozygous, and Cre transgenic animals without lox sites (CON, HET, KO Cre+). Rec signal is reduced in CON and HET versus KO. **d** RT-PCR analysis of titin M-band exon 1-2 (*MEx1-2*), and C-terminus (*MEx6*) in control, heterozygous, and knockout mice with a significant and strong reduction of mRNA encoding titin's kinase region (*MEx1-2*) in knockout animals. Mean with SEM; n = 3 per group, Two-way ANOVA followed by Bonferroni's post hoc test was performed to test differences between CON and KO groups** $p < 0.005$

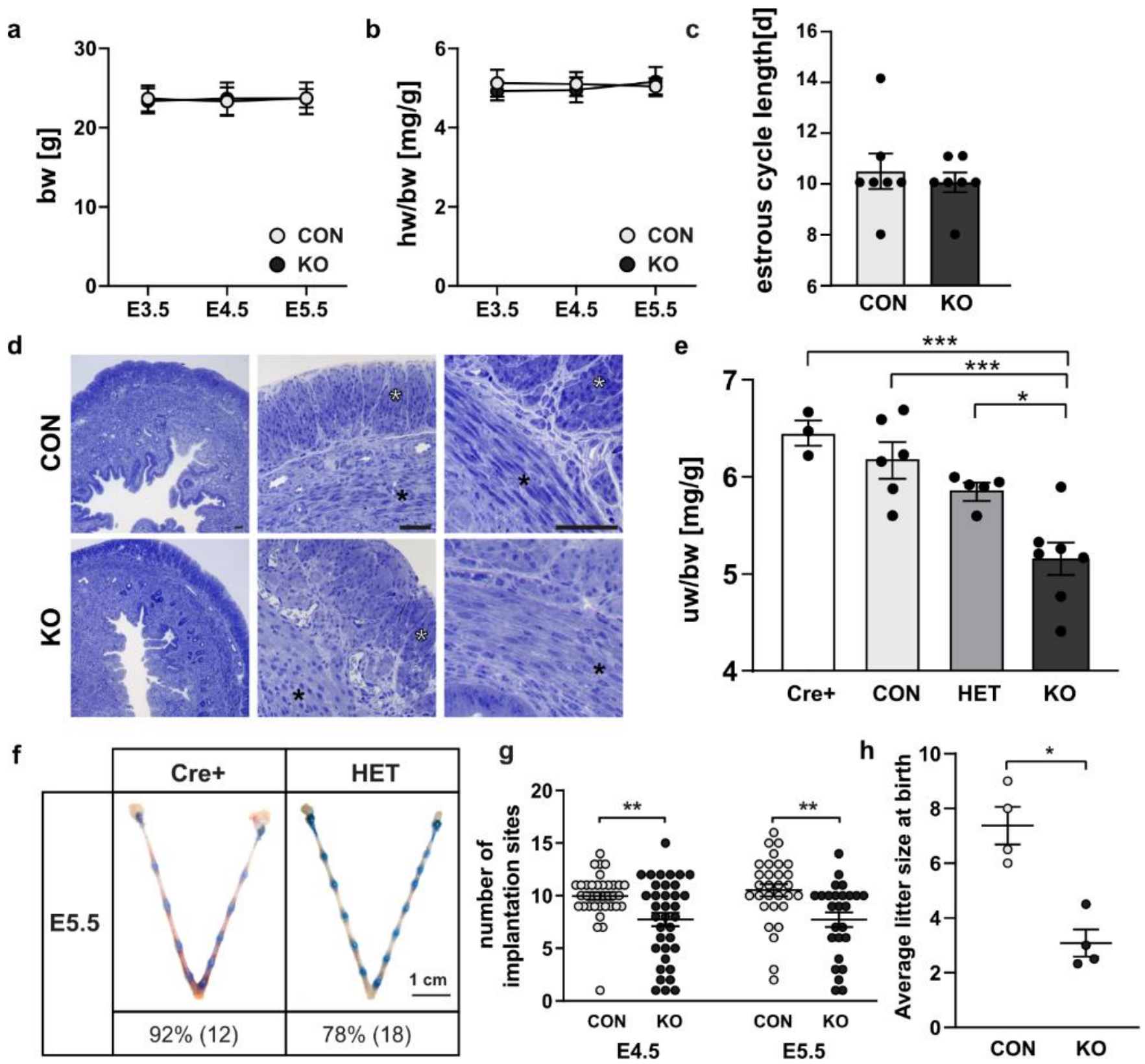

Figure S2: Impaired uterine development in normotrophic mice was independent of the presence of the Cre-recombinase. **a, b** Bodyweight (bw) and heart to bodyweight ratio (hw/bw) were not altered in smooth muscle knockout females around the time of implantation (Embryonic day E3.5 to 5.5). **c** Estrous cycle length did not differ between knockout (KO) and controls (CON). **d** Normal morphology of CON and KO uterus. Asterisks indicate the myometrium (white - longitudinal, black - circular muscle). **e** Uterus weight per body weight (uw/bw) at E5.5 was significantly reduced in KO females but not in control mice carrying the cre allele (Cre+) or one mutant titin allele (HET). **f** Normal implantation was documented in ~80-90% of HET and Cre+ females. **g** The number of implantation sites was significantly reduced in knockout mothers. **h** Average litter size at birth was significantly reduced in knockout (n=4) Size bar = 50 µm. CON n = 6; HET n = 5; KO n = 6; Cre+ n = 3; One-way ANOVA (a, e), Student's t-test (c), Welch`s t-test (h), Two-way ANOVA (g) with Tukey's *post hoc* tests was performed to test differences between CON and KO groups* $p < 0.05$, ** $p < 0.01$, *** $p < 0.001$

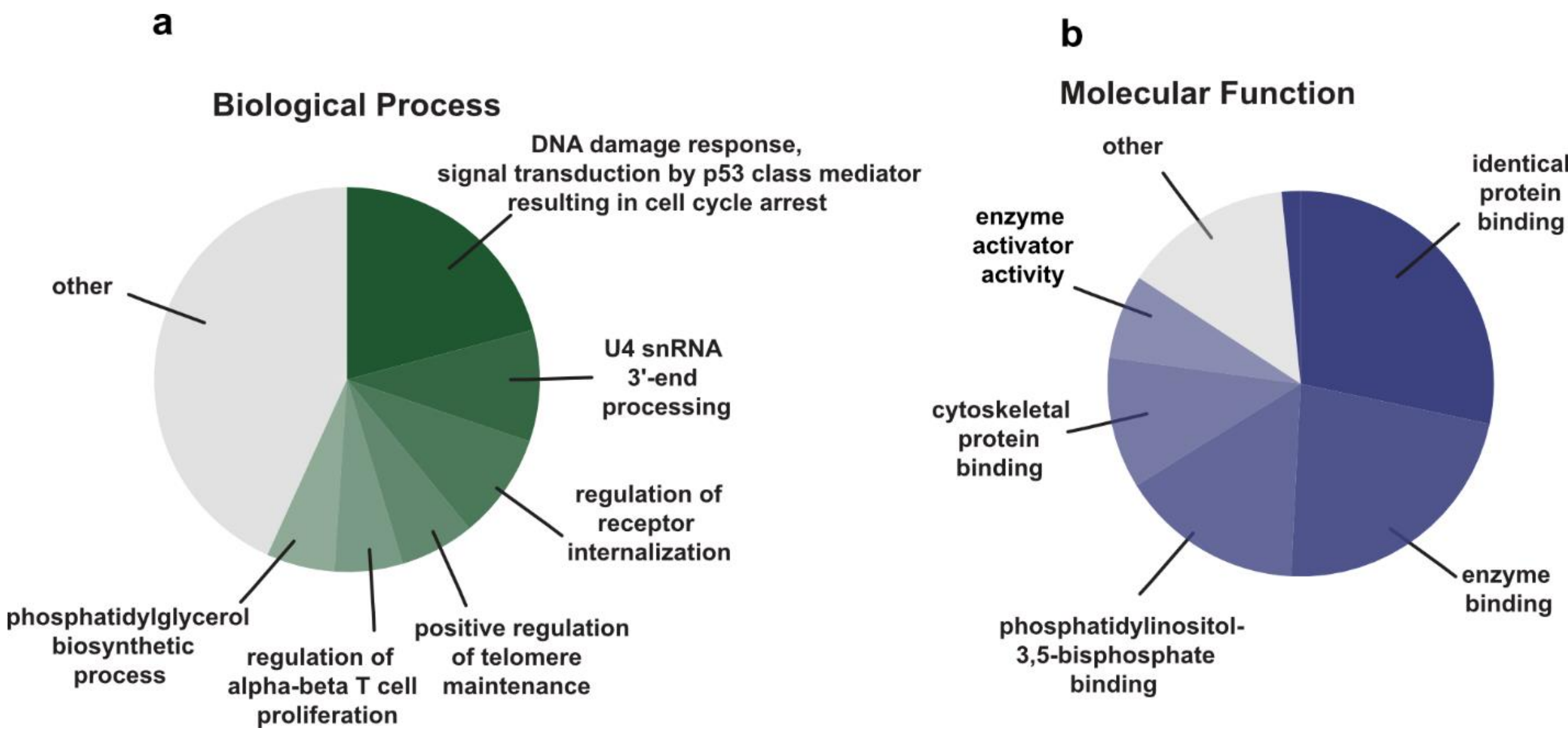

Figure S3: Transcriptional changes in the titin deficient uterus. Gene ontology analysis for Biological Process and Molecular Function. Transcripts altered in the pregnant smooth muscle titin KO uterus relate to proliferation and protein/protein interaction.

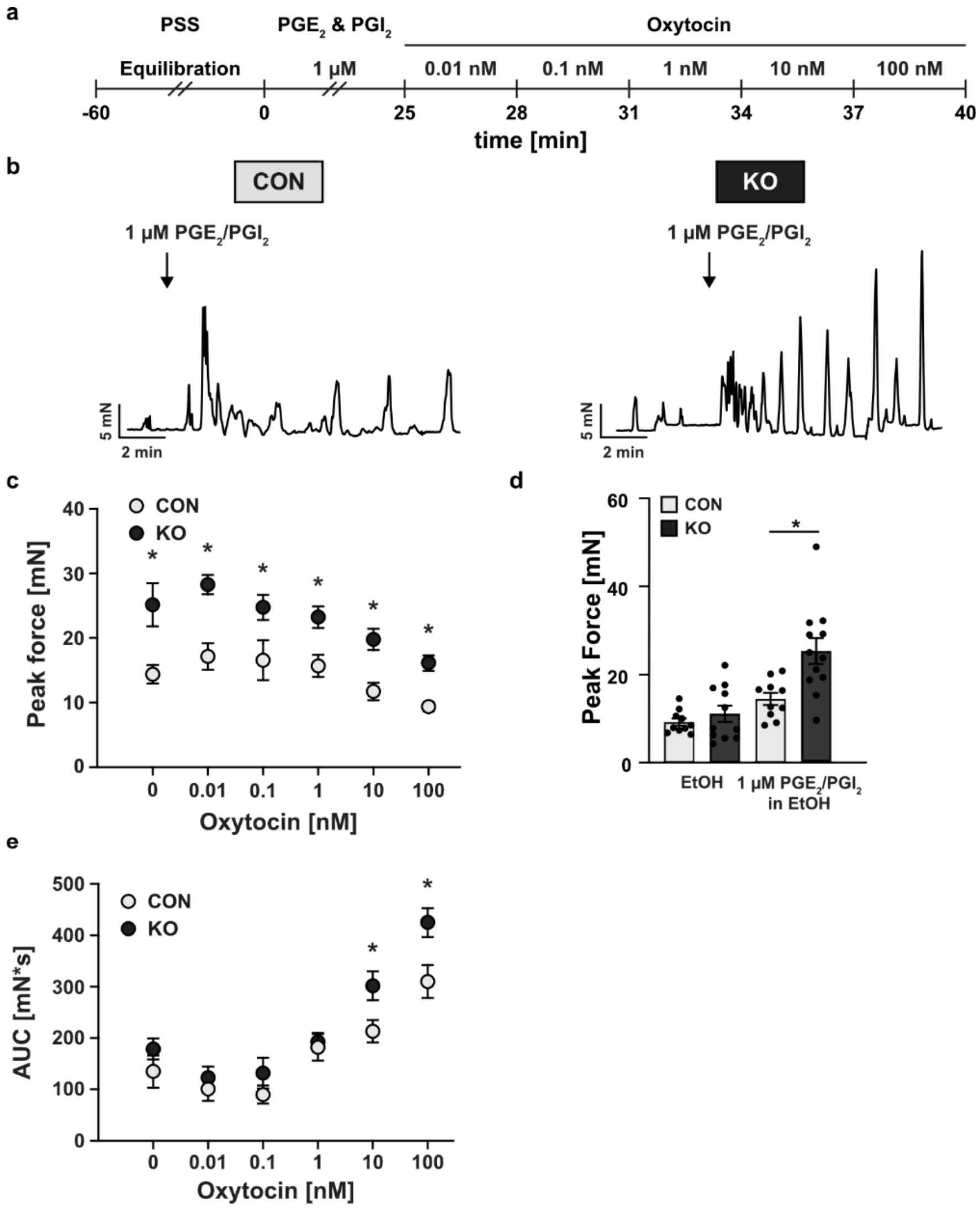

Figure S4: Reduced Oxytocin response to prostaglandin in titin KO uterus. **a** Experimental protocol with 60 min equilibration, 25 min prostaglandin treatment (1 µM PGE2 and PGI2) and subsequent treatment with increasing oxytocin concentrations (0.01 nM - 100 nM). **b** original traces of force response in uterus rings from titin smooth muscle control (CON) and KO-mice. **c,d** Peak force response to oxytocin after PGE treatment was increased, as was the baseline response (**c**). Ethanol (EtOH) was the vehicle control. **e** Area under curve (AUC) was increased at higher oxytocin concentrations in the knockout uterus. N = 10-12 rings derived from 3 animals per genotype; Two-way ANOVA with Tukey *post hoc* tests was performed to test differences between CON and KO groups* $p < 0.05$

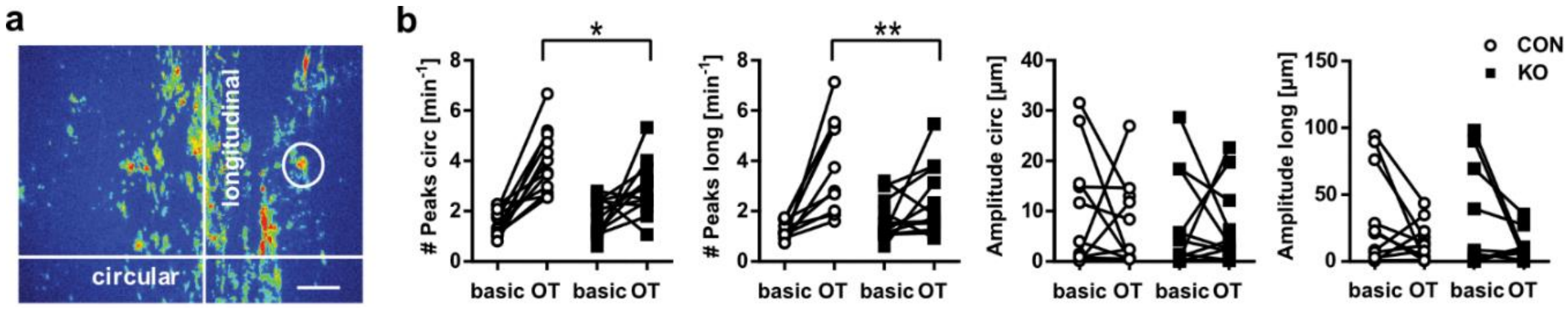

Figure S5: Reduced wall movement in titin deficient uterus in response to oxytocin (OT). **a** longitudinally opened uterus wall to expose the circular and longitudinal muscle layers. The white circle surrounds a signal that was tracked. Size bar = 200 µm. **b** Individual data points from figure 5b comparing number of peaks (# Peaks) and amplitudes for movement along the longitudinal axis (long) or the circular muscle layer (circ). Two-way ANOVA followed by Bonferroni's post hoc test was performed to test differences between CON and KO groups* $p < 0.05$, ** $p < 0.01$

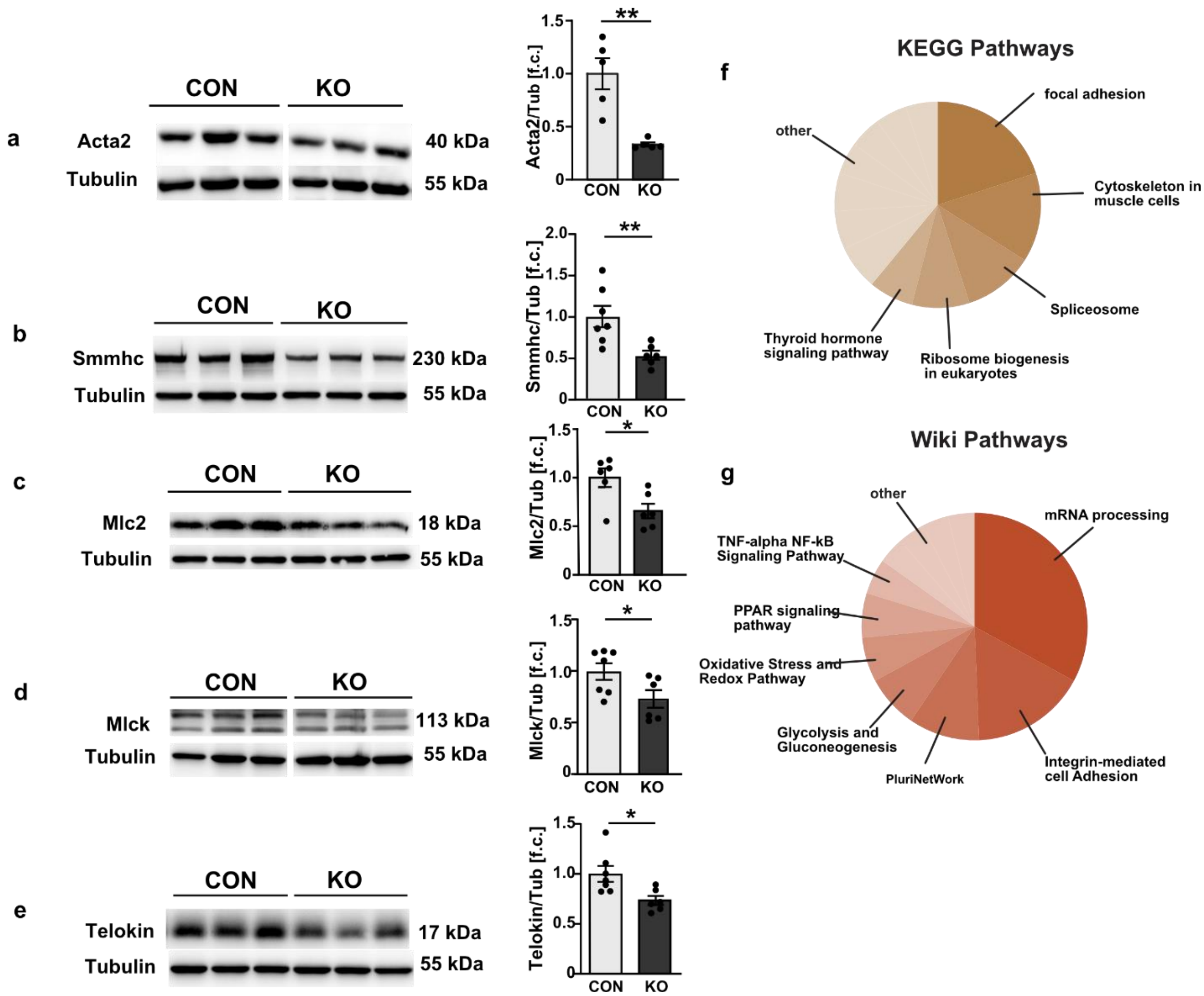

Figure S6: Proteins involved in smooth muscle contraction were deregulated in M-band titin deficient uterus at E3.5. Expression normalized to tubulin. Blot left, quantification right. Non-adjacent lanes from the same blot are separated by a space. a, b alpha- actin 2 (Acta2) and smooth muscle myosin heavy chain (Smmhc) levels were downregulated Myosin light chain kinase (Mylk) c, myosin light chain (Mlc2) d, and telokin were downregulated on the protein level (quantification right) n = 5-7 animals per genotype; Student's t test; * $p < 0.05$, ** $p < 0.01$

Tables

Table S1: Smooth muscle titin proteomics.

The MS data is summarized in a separate excel file (Table S1 Peptides.xlsx)

Table S2: Extended KEGG pathway enrichment analysis

GOID	GOTerm	Nr. Genes	%	Associated Genes
KEGG:05203	Viral carcinogenesis	11	4.8	[Cdkn1a, Chd4, Hist1h2bf, Hist1h2bh, Mad1l1, Mapkapk2, Mdm2, Rb1, Snd1, Snw1, Traf1]
KEGG:04650	Natural killer cell mediated cytotoxicity	7	5.9	[Hcst, Klra4, Klra7, Klrd1, Lck, Nfatc1, Rac3]
KEGG:05166	HTLV-I infection	7	2.5	[Cdkn1a, Chek2, H2-Ob, Lck, Nfatc1, Rb1, Slc25a5]
KEGG:05202	Transcriptional misregulation in cancer	7	4.0	[Cdkn1a, Etv5, Fus, Igfbp3, Lyl1, Mdm2, Traf1]
KEGG:04921	Oxytocin signaling pathway	7	4.6	[Camk2a, Cdkn1a, Eef2k, Itpr2, Myl6, Nfatc1, Rock1]
KEGG:04510	Focal adhesion	7	3.5	[Egf, Hgf, Itga1, Itgb8, Rac3, Rapgef1, Rock1]
KEGG:03018	RNA degradation	6	7.4	[Dcps, Eno3, Exosc5, Exosc6, Lsm4, Skiv2l2]
KEGG:04066	HIF-1 signaling pathway	6	5.6	[Camk2a, Cdkn1a, Egf, Eif4e2, Eno3, Ldha]
KEGG:04142	Lysosome	6	4.8	[Ctse, Ctsk, Ctsw, Dnase2a, Gm2a, Hyal1]
KEGG:04144	Endocytosis	6	2.1	[Egf, Ehd1, Gbf1, Grk6, Mdm2, Mvb12a]
KEGG:04115	p53 signalling pathway	6	8.8	[Cdkn1a, Chek2, Igfbp3, Mdm2, Shisa5, Zmat3]

Table S3: Wiki pathway enrichment analysis

GOID	GOTerm	Nr. Genes	%	Associated Genes
WP:2309	XPodNet - protein-protein interactions in the podocyte expanded by STRING	17	2.0	[Angptl4, Bmp4, Camk2a, Cdkn1a, Epha4, Fyb, Hand2, Igfbp3, Itga1, Lck, Lims2, Mdm2, Pdxp, Ptprc, Rab27a, Sema3a, Snta1]
WP:1763	PluriNetWork	11	3.7	[Bmp4, Cdkn1a, Chd4, Ctr9, Etv5, Hand2, Igfbp3, Kpnb1, Mdm2, Rock1, Tpo]
WP:2841	Focal Adhesion-PI3K-Akt-mTOR-signaling pathway	8	2.5	[Cdkn1a, Egf, Eif4e2, Ghr, Gng8, Hgf, Itgb8, Mdm2]
WP:310	mRNA processing	7	1.5	[Dhx8, Eif4e2, Exosc5, Fus, Lsm4, Phf5a, Rpl22]
WP:1251	Metapathway biotransformation	6	4.3	[Ephx1, Fmo1, Hs3st3a1, Inmt, Mgst2, Mgst3]
WP:385	Myometrial Relaxation and Contraction Pathways	6	3.8	[Camk2a, Gng8, Grk6, Igfbp3, Itpr2, Rgs1]
WP:6	Integrin-mediated Cell Adhesion	6	6.1	[Itga1, Itgb8, Rac3, Rapgef1, Rock1, Selenop]
WP:2902	p53 signaling	6	9.0	[Cdkn1a, Chek2, Igfbp3, Mdm2, Shisa5, Zmat3]
WP:246	TNF-alpha NF-kB Signaling Pathway	5	2.6	[Csnk2a2, Fkbp5, Psmd12, Tnip1, Traf1]

Table S4: Biological process enrichment analysis

GOID	GOTerm	Nr. Genes	%	Associated Genes
GO:0072332	intrinsic apoptotic signaling pathway by p53 class mediator	8	9.2	[Cdkn1a, Chek2, Hint1, Mdm2, Phlda3, Shisa5, Snw1, Zmat3]
GO:0007173	epidermal growth factor receptor signaling pathway	6	6.4	[Caml, Efemp1, Egf, Hip1, Itga1, Mvb12a]
GO:0042130	negative regulation of T cell proliferation	5	8.6	[Bmp4, Cd276, Mad1l1, Tnfrsf14, Xcl1]
GO:0060795	cell fate commitment involved in formation of primary germ layer	4	12.5	[Bmp4, Ctr9, Elf5, Sfrp2]
GO:1902041	regulation of extrinsic apoptotic signaling pathway via death domain receptors	4	7.4	[Hgf, Mkl1, Pea15a, Sfrp2]
GO:0030834	regulation of actin filament depolymerization	4	7.7	[Pdxp, Tmod3, Twf2, Wdr1]
GO:0010665	regulation of cardiac muscle cell apoptotic process	4	12.5	[Camk2a, Eif5a, Hand2, Sfrp2]
GO:1904356	regulation of telomere maintenance via telomere lengthening	4	7.5	[Acd, Slx4, Stn1, Tnip1]
GO:0008038	neuron recognition	3	9.1	[Epha4, Gap43, Sema3a]
GO:0038066	p38MAPK cascade	3	8.6	[Hand2, Hgf, Mapkapk2]
GO:0043489	RNA stabilization	3	7.9	[Mapkapk2, Noct, Tnip1]
GO:0003179	heart valve morphogenesis	3	7.7	[Bmp4, Mdm2, Nfatc1]
GO:0060071	Wnt signaling pathway, planar cell polarity pathway	3	8.1	[Ptk7, Ror2, Sfrp2]
GO:0002335	mature B cell differentiation	3	12.5	[Gpr183, Malt1, Nfatc1]
GO:0019674	NAD metabolic process	3	7.9	[Idh3b, Ldha, Nadk]
GO:0030225	macrophage differentiation	3	9.1	[Bmp4, Rb1, Ror2]

GO:0035413	positive regulation of catenin import into nucleus	3	25.0	[Egf, Kank1, Sfrp2]
GO:1901028	regulation of mitochondrial outer membrane permeabilization involved in apoptotic signaling pathway	3	15.8	[Atpif1, Gm5560, Slc25a5]
GO:0006655	phosphatidylglycerol biosynthetic process	3	18.8	[Chek2, Slc27a1, Tamm41]
GO:0001731	formation of translation preinitiation complex	2	12.5	[Eif3h, Mcts1]
GO:0044791	positive regulation by host of viral release from host cell	2	28.6	[Vapa, Zfp87]
GO:0045588	positive regulation of gamma-delta T cell differentiation	2	33.3	[Lck, Ptprc]
GO:0048133	male germ-line stem cell asymmetric division	2	50.0	[Etv5, Ing2]
GO:0051571	positive regulation of histone H3-K4 methylation	2	13.3	[Ctr9, Snw1]
GO:0071072	negative regulation of phospholipid biosynthetic process	2	50.0	[Lpcat1, Slc27a1]
GO:0090324	negative regulation of oxidative phosphorylation	2	33.3	[Dnajc15, Mlxipl]
GO:0010792	DNA double-strand break processing involved in repair via single-strand annealing	2	50.0	[Rad52, Slx4]
GO:0034030	ribonucleoside bisphosphate biosynthetic process	2	16.7	[Papss1, Ppcs]
GO:0061325	cell proliferation involved in outflow tract morphogenesis	2	40.0	[Bmp4, Hand2]
GO:0034475	U4 snRNA 3'-end processing	2	40.0	[Exosc5, Exosc6]

Table S5: Extended molecular function enrichment analysis

GOID	GOTerm	Nr. Genes	%	Associated Genes
GO:0019899	enzyme binding	57	3.0	[Acd, Als2, Amfr, Atpif1, Btbd9, C1qbp, Camk2n1, Cat, Cdkn1a, Chd4, Chek2, Ctnnbl1, Ctr9, Dbt, Ehd1, Eif4e2, Fiz1, Ghr, Gm5560, Ipo5, Itga1, Kif3a, Klre1, Kpnb1, Lck, Ldha, Malt1, Map3k12, Mdm2, Myo5b, Myrip, Nfatc1, Nkain1, Nsf, Nutf2, Parp16, Pea15a, Ppp1r26, Ppp6r3, Prdm4, Prkcsh, Ptprc, Rb1, Rnf185, Rock1, Slc25a5, Snta1, Stbd1, Tbc1d10c, Tbc1d13, Tnfrsf14, Tnip1, Tor1aip2, Traf1, Tspan33, Twf2, Zmynd15]
GO:0042802	identical protein binding	48	3.3	[Alas1, Als2, Angptl4, Apip, Atpif1, Bst2, Camk2a, Cat, Chek2, Coil, Coro1a, Ctse, Des, Dhx8, Dnttip1, Dynlrb1, Eno3, Epha4, Fgfr1op2, Fnbp1, Fus, Ghr, Gimap7, Hand2, Hgf, Hip1, Hsf2, Hspb8, Khdc1c, Klre1, Kyat1, Lck, Ldha, Map3k12, Mcm6, Mdm2, Mlxipl, Nde1, Oat, Papss1, Pdxp, Rad52, Rb1, Slc27a1, Tnip1, Traf1, Uap1, Xcl1]
GO:0008092	cytoskeletal protein binding	28	3.3	[Actr3b, Ankrd23, Chd4, Coro1a, Dctn1, Des, Epb41, Eps8l1, Fus, Gphn, Hip1, Ino80, Kif27, Kif3a, Krt10, Mkl1, Myo5b, Myrip, Nde1, Rab27a, Snta1, Tmod3, Tmsb10, Trim63, Tubgcp4, Twf2, Vapa, Wdr1]
GO:0008047	enzyme activator activity	18	4.2	[Als2, Arhgap18, Cdkn1a, Depdc5, Dnttip1, Gm2a, Guca1a, Igfbp3, Malt1, Mgst2, Parp16, Pgam5, Rgs1, Sfrp2, Slx4, Tbc1d10c, Tbc1d13, Tor1aip2]
GO:0035091	phosphatidylinositol binding	13	6.0	[Atp13a2, Epb41, Gap43, Gbf1, Hip1, Hs1bp3, Ing2, Itpr2, Mapkapk2, Mark1, Phlda3, Tub, Twf2]
GO:0004721	phosphoprotein phosphatase activity	8	4.8	[Ctdspl, Lck, Pdp2, Pdxp, Pgam5, Ptpn9, Ptprc, Ublcp1]
GO:0017137	Rab GTPase binding	7	5.4	[Als2, Ehd1, Kif3a, Myo5b, Myrip, Tbc1d10c, Tbc1d13]
GO:0051117	ATPase binding	5	6.6	[Atpif1, Lck, Nkain1, Snta1, Tor1aip2]
GO:0030170	pyridoxal phosphate binding	5	9.4	[Alas1, Gpt, Hdc, Kyat1, Oat]

GO:0003785	actin monomer binding	4	14.8	[Coro1a, Mkl1, Tmsb10, Twf2]
GO:0004520	endodeoxyribonuclease activity	4	7.8	[Dnase2a, Nthl1, Slx4, Smug1]
GO:0004601	peroxidase activity	4	9.1	[Cat, Mgst2, Mgst3, Tpo]
GO:0019899	enzyme binding	57	3.0	[Acd, Als2, Amfr, Atpif1, Btbd9, C1qbp, Camk2n1, Cat, Cdkn1a, Chd4, Chek2, Ctnnbl1, Ctr9, Dbt, Ehd1, Eif4e2, Fiz1, Ghr, Gm5560, Ipo5, Itga1, Kif3a, Klre1, Kpnb1, Lck, Ldha, Malt1, Map3k12, Mdm2, Myo5b, Myrip, Nfatc1, Nkain1, Nsf, Nutf2, Parp16, Pea15a, Ppp1r26, Ppp6r3, Prdm4, Prkcsh, Ptprc, Rb1, Rnf185, Rock1, Slc25a5, Snta1, Stbd1, Tbc1d10c, Tbc1d13, Tnfrsf14, Tnip1, Tor1aip2, Traf1, Tspan33, Twf2, Zmynd15]
GO:0042802	identical protein binding	48	3.3	[Alas1, Als2, Angptl4, Apip, Atpif1, Bst2, Camk2a, Cat, Chek2, Coil, Coro1a, Ctse, Des, Dhx8, Dnttip1, Dynlrb1, Eno3, Epha4, Fgfr1op2, Fnbp1, Fus, Ghr, Gimap7, Hand2, Hgf, Hip1, Hsf2, Hspb8, Khdc1c, Klre1, Kyat1, Lck, Ldha, Map3k12, Mcm6, Mdm2, Mlxipl, Nde1, Oat, Papss1, Pdxp, Rad52, Rb1, Slc27a1, Tnip1, Traf1, Uap1, Xcl1]
GO:0008092	cytoskeletal protein binding	28	3.3	[Actr3b, Ankrd23, Chd4, Coro1a, Dctn1, Des, Epb41, Eps8l1, Fus, Gphn, Hip1, Ino80, Kif27, Kif3a, Krt10, Mkl1, Myo5b, Myrip, Nde1, Rab27a, Snta1, Tmod3, Tmsb10, Trim63, Tubgcp4, Twf2, Vapa, Wdr1]
GO:0008047	enzyme activator activity	18	4.2	[Als2, Arhgap18, Cdkn1a, Depdc5, Dnttip1, Gm2a, Guca1a, Igfbp3, Malt1, Mgst2, Parp16, Pgam5, Rgs1, Sfrp2, Slx4, Tbc1d10c, Tbc1d13, Tor1aip2]

Table S6: Wiki pathway enrichment analysis (proteomics)

GOID	GOTerm	Nr. Genes	%	Associated Genes
WP:157	Glycolysis and Gluconeogenesis	6.00	11.76	[Eno1, Gpi1, Pfkp, Pgam1, Pgk1, Pkm]
WP:163	Cytoplasmic Ribosomal Proteins	3.00	2.91	[Rpl35, Rps18, Rps28]
WP:310	mRNA processing	26.00	5.65	[Acin1, Cpsf2, Csad, Ddx21, Ddx24, Ddx5, Ddx56, Dhx9, Nono, Nxf1, Oasl2, Papola, Pum3, Raly, Rbm17, Rps28, Rps9, Scaf8, Sf3a3, Sf3b1, Sf3b2, Snrnp70, Srsf5, Sugp1, Tnrc6c, Tut1]
WP:4466	Oxidative Stress and Redox Pathway	5.00	5.32	[Cybb, Keap1, Lap3, Slco2a1, Sod2]
WP:523	Regulation of Actin Cytoskeleton	3.00	1.97	[Arpc5, Rock1, Vcl]
WP:567	Eukaryotic Transcription Initiation	3.00	6.98	[Cdk7, Ilk, Polr1a]
WP:1763	PluriNetWork: mechanisms associated with pluripotency	8.00	2.72	[Chd4, Ddb1, Dhx9, Gatad2a, Itgb1, Rock1, Sf1, Zfp219]
WP:2185	Purine metabolism	3.00	1.75	[Adk, Pkm, Polr1a]
WP:2316	PPAR signaling pathway	5.00	6.17	[Fads2, Ilk, Me1, Pltp, Sorbs1]
WP:246	TNF-alpha NF-kB Signaling Pathway	4.00	2.12	[Akap8, Cav1, Papola, Polr1a]
WP:2573	Primary Focal Segmental Glomerulosclerosis FSGS	5.00	6.94	[Ilk, Itgb1, Itgb3, Tln1, Vcl]

WP:2841	Focal Adhesion-PI3K-Akt-mTOR-signaling pathway	5.00	1.54	[Acaca, Itgb1, Itgb3, Lama4, Lamb1]
WP:3675	Factors and pathways affecting insulin-like growth factor (IGF1)-Akt signaling	3.00	9.68	[Igfbp7, Ilk, Itgb1]
WP:6	Integrin-mediated Cell Adhesion	9.00	9.00	[Cav1, Ilk, Itgb1, Itgb3, Rock1, Sorbs1, Tln1, Tns1, Vcl]
WP:85	Focal Adhesion	9.00	4.86	[Cav1, Ilk, Itgb1, Itgb3, Lama4, Lamb1, Rock1, Tln1, Vcl]

Table S7: Extended KEGG pathway enrichment analysis (proteomics)

GOID	GOTerm	Nr. Genes	%	Associated Genes
KEGG:00010	Glycolysis / Gluconeogenesis	6.00	8.96	[Eno1, Gpi1, Pfkp, Pgam1, Pgk1, Pkm]
KEGG:03008	Ribosome biogenesis in eukaryotes	9.00	7.20	[Bms1, Dkc1, Lsg1, Nat10, Nxf1, Tcof1, Utp4, Wdr3, Wdr36]
KEGG:04141	Protein processing in endoplasmic reticulum	5.00	2.86	[Amfr, Dnajb1, Edem3, Hspbp1, Hsph1]
KEGG:04621	NOD-like receptor signaling pathway	5.00	2.31	[Cybb, Dhx33, Pycard, Txnip, Vdac1]
KEGG:04820	Cytoskeleton in muscle cells	14.00	6.03	[Atp1a1, Atp1a2, Capzb, Csrp1, Eno1, Fhl1, Flnc, Itgb1, Itgb3, Lmnb1, Pdlim7, Sun2, Tln1, Vcl]
KEGG:05164	Influenza A	5.00	2.87	[Dnajb1, Nup98, Nxf1, Pycard, Vdac1]
KEGG:05171	Coronavirus disease	7.00	2.10	[Cybb, Fcgr3, Mmp3, Rpl35, Rps18, Rps28, Rps9]
KEGG:03040	Spliceosome	11.00	4.15	[Acin1, Aqr, Ddx42, Ddx5, Rbm17, Sf3a3, Sf3b1, Sf3b2, Snrnp70, Srsf5, Tcerg1]
KEGG:03320	PPAR signaling pathway	6.00	6.74	[Fads2, Hmgcs1, Ilk, Me1, Pltp, Sorbs1]
KEGG:04120	Ubiquitin mediated proteolysis	5.00	3.25	[Anapc2, Cul7, Ddb1, Keap1, Trip12]
KEGG:04022	cGMP-PKG signaling pathway	5.00	2.91	[Atp1a1, Atp1a2, Atp2a3, Rock1, Vdac1]
KEGG:04919	Thyroid hormone signaling pathway	5.00	4.17	[Atp1a1, Atp1a2, Atp2a3, Itgb3, Pfkp]
KEGG:04145	Phagosome	5.00	2.73	[Cybb, Fcgr3, Itgb1, Itgb3, Mrc2]
KEGG:04510	Focal adhesion	10.00	4.95	[Cav1, Ilk, Itgb1, Itgb3, Lama4, Lamb1, Ppp1r12c, Rock1, Tln1, Vcl]

KEGG:04611	Platelet activation	5.00	3.97	[Fcgr3, Itgb1, Itgb3, Rock1, Tln1]
KEGG:04810	Regulation of actin cytoskeleton	6.00	2.59	[Arpc5, Itgb1, Itgb3, Ppp1r12c, Rock1, Vcl]
KEGG:05100	Bacterial invasion of epithelial cells	5.00	6.49	[Arpc5, Cav1, Ilk, Itgb1, Vcl]
KEGG:05135	Yersinia infection	5.00	3.68	[Arpc5, Fcgr3, Itgb1, Pycard, Rock1]
KEGG:05165	Human papillomavirus infection	7.00	1.95	[Chd4, Itgb1, Itgb3, Lama4, Lamb1, Oasl2, Pkm]
KEGG:05205	Proteoglycans in cancer	7.00	3.43	[Cav1, Ddx5, Itgb1, Itgb3, Nudt16l1, Ppp1r12c, Rock1]

Table S8: Primers and TaqMan probes

Name	Sequence	Used for
18S RNA fw	CGCCGCTAGAGGTGAAATTC	RT-qPCR
18S RNA rev	TGGGCAAATGCTTTCGCTC	RT-qPCR
18S RNA probe	6-FAM-TGGACCGGCGCAAGACGGAC-TAMRA	RT-qPCR
MEx1-2 fw	CCGATGGACTCAAGTACAGGATT	RT-qPCR
MEx1-2 rev	CCCATGCCTTCGAGAGTCTT	RT-qPCR
MEx1-2 probe	6-FAM-TCCTTGGAAGTGGAAGTTCCAGCTAAGATACAC-TAMRA	RT-qPCR
MEx6 fw	GCCTTGTGTGGTAGTTCTAAATTCAA	RT-qPCR
MEx6 rev	TTTGCTGTGGCTCATTGCTT	RT-qPCR
MEx6 probe	6-FAM-TTTCACCGGGAACTGGGCAA-TAMRA	RT-qPCR
SMMHCcre-for	CCAATTTACTGACCGTACACC	Genotyping
SMMHCcre-rev	GTTTCACTATCCAGGTTACGG	Genotyping
Ti-SL1	GTGTCTGGCACTGCTTCCTTGGAAGTG	Genotyping
Ti-SL2	ACCGCTCCCATGCCTTCGAGAGTCTTG	Genotyping
Ti-FRTr2	AAGTTCGCTATACAACTGAGGCTAAG	Genotyping

Primer/probes for Real-Time PCR analysis provided in 5’ to 3’orientation (fw: forward; rev: reverse; FAM: 6-carboxyfluorescein; TAMRA: 6-carboxytetramethylrhodamine).

Table S9: Antibodies

Antigen	Species	Company	Antibody
Acta2	Mouse	Sigma-Aldrich	A2547
Beta-Tubulin	Rabbit	Cell Signaling	2146
Mlc2	Rabbit	Cell signaling	8505
MuRF1	Rabbit	Abcam	ab77577
Mylk	Rabbit	Abcam	ab76092
Nbr1	Rabbit	Cell signaling	9891
p62	Mouse	Abcam	ab56416
Smmhc	Rabbit	Abcam	ab53219

Data S1: SDS and western blot images

Figure 1 Gels

Uterus Qa (Controls)

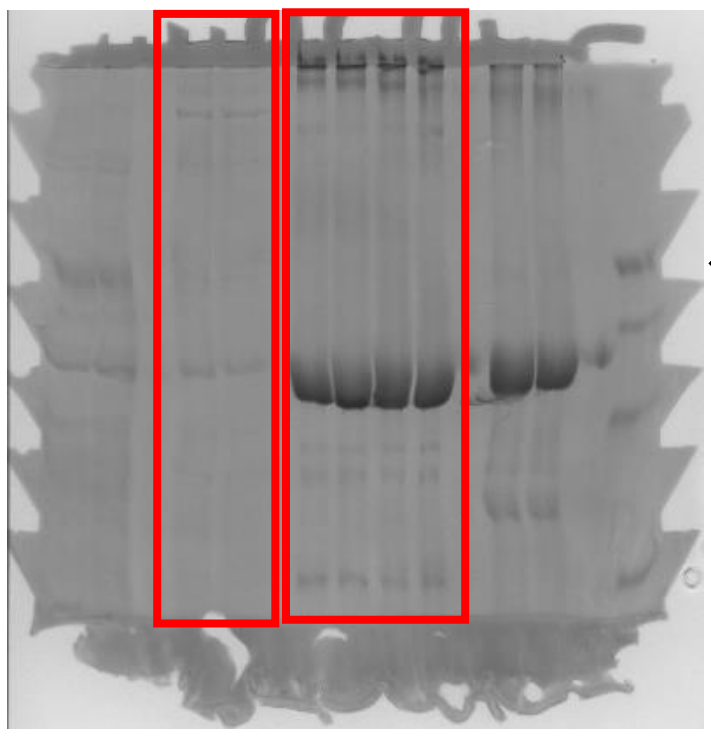

300 kDa

Uterus Qa (control)

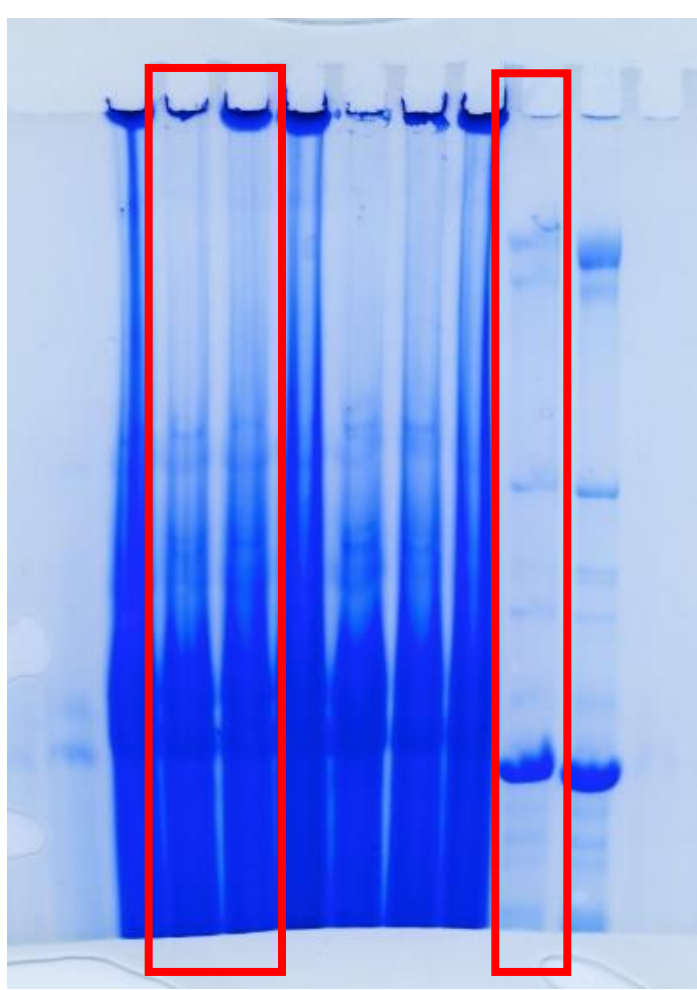

Figure 6 b

NBR1

CON KO

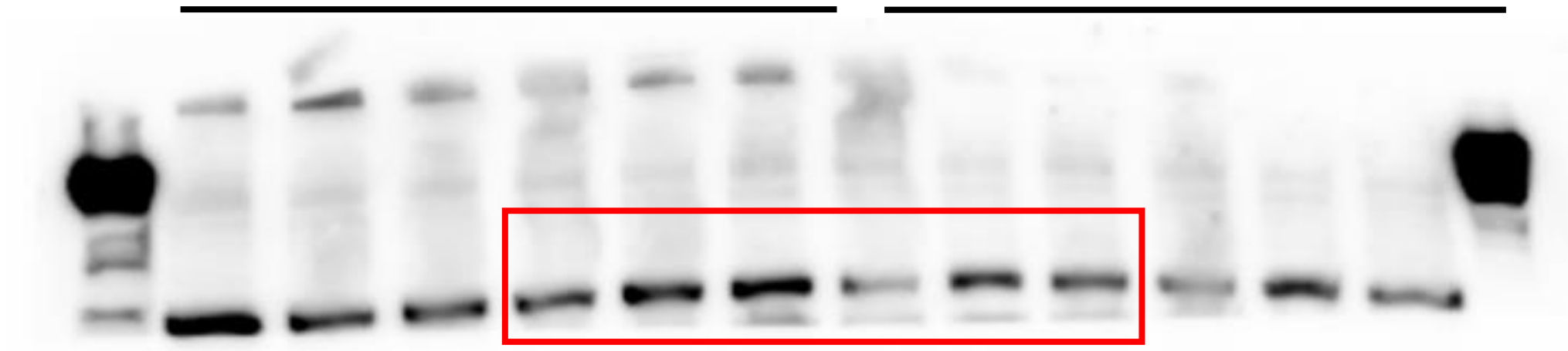

130 kDa

B-tubulin

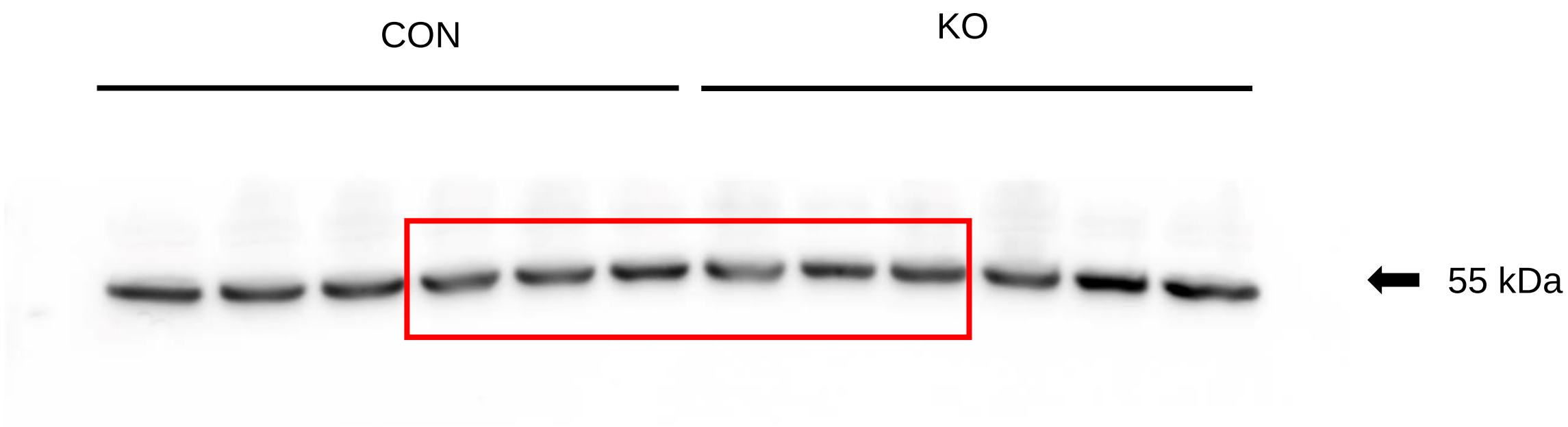

MuRF1

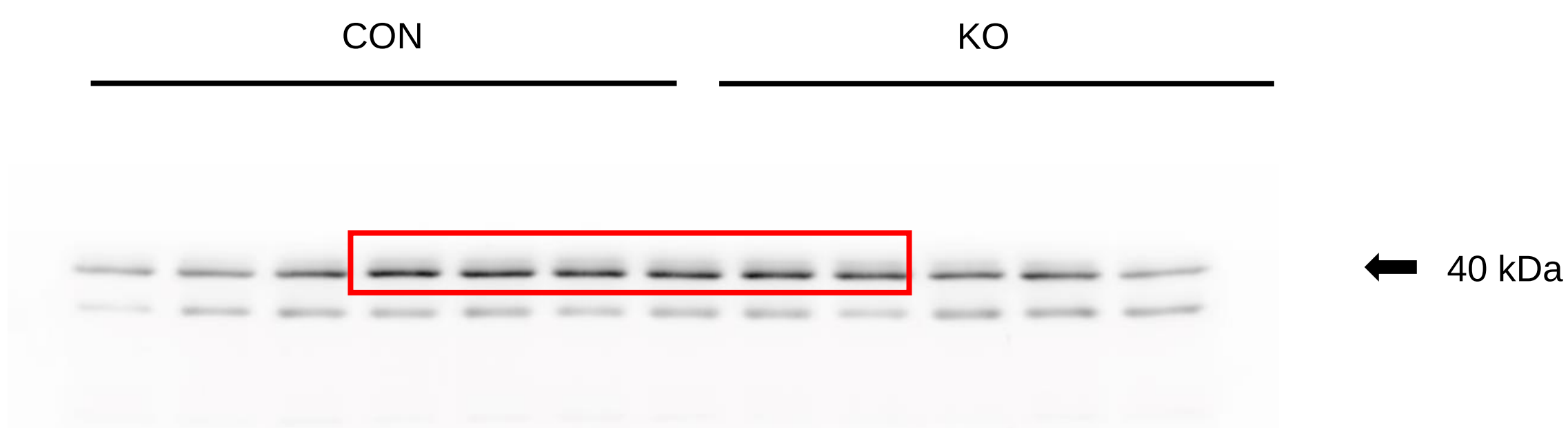

B-tubulin

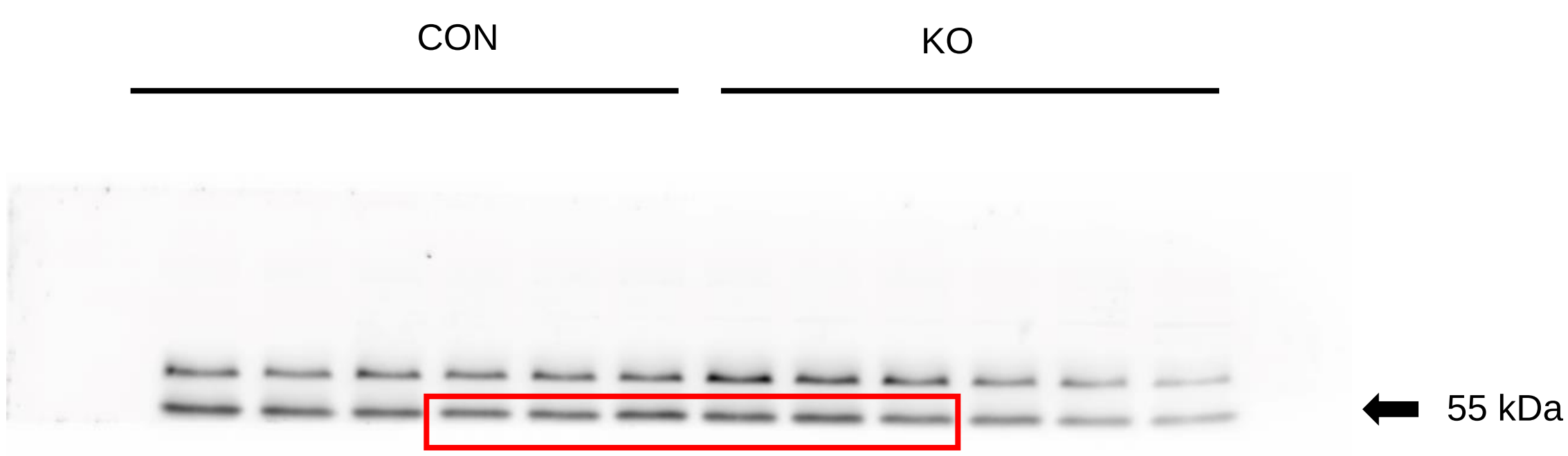

P62

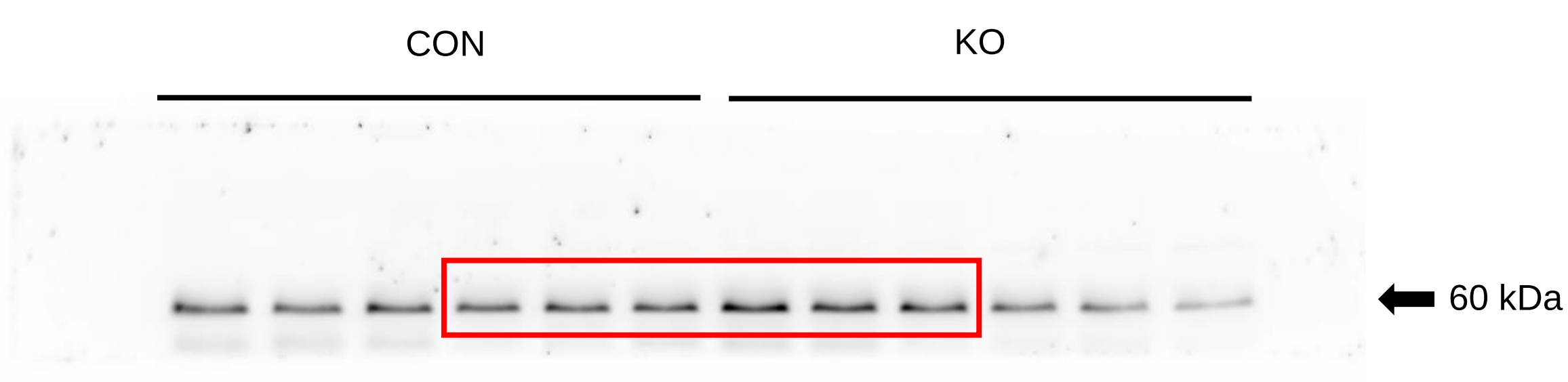

B-tubulin

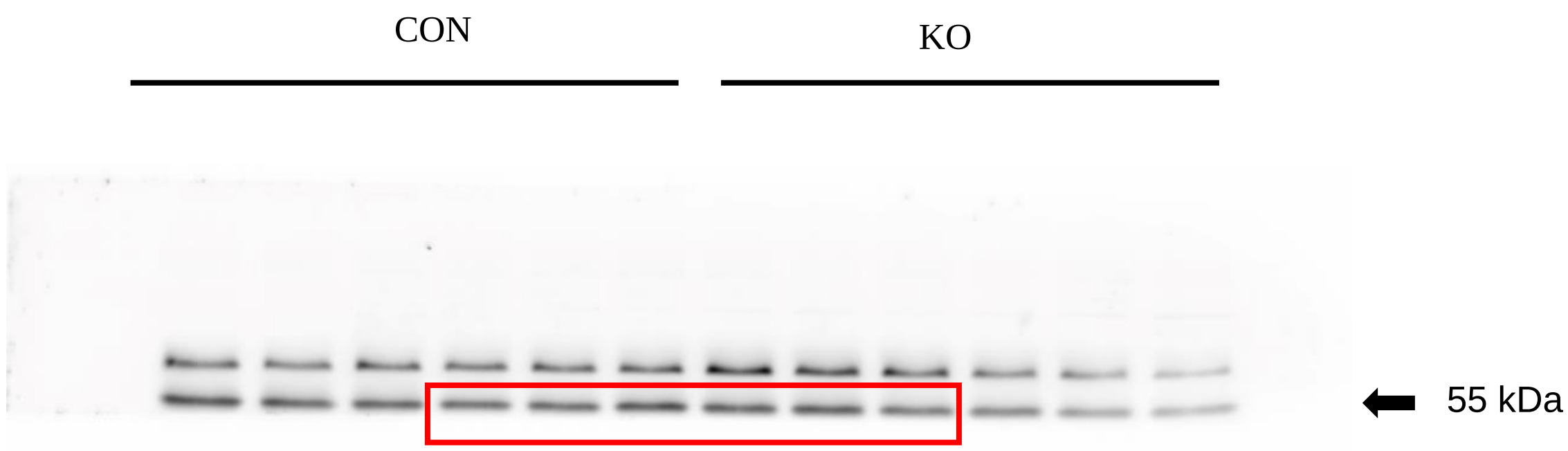

Figure S6a Acta2

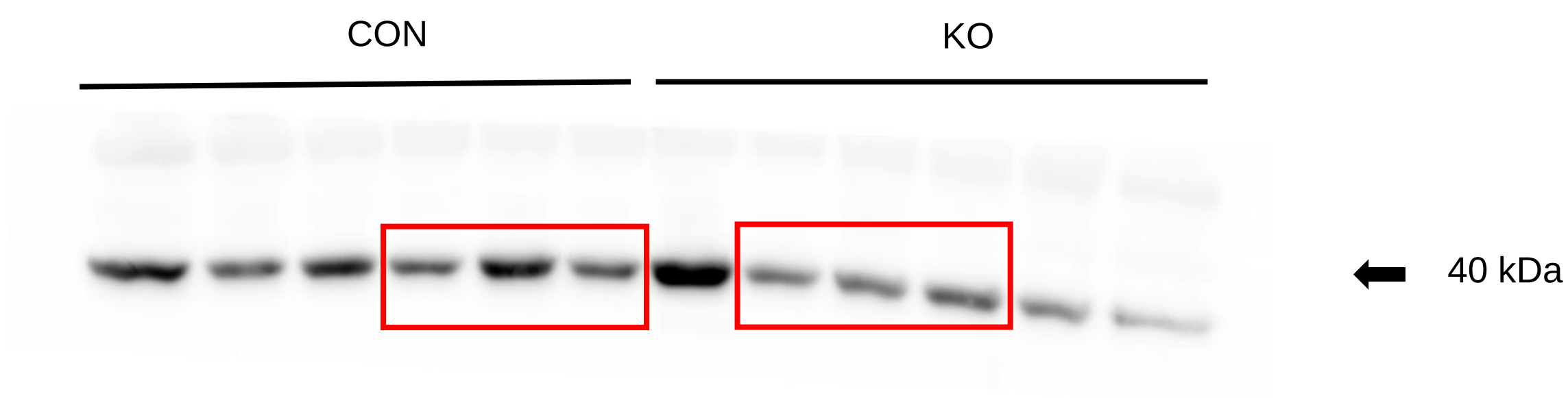

B-tubulin

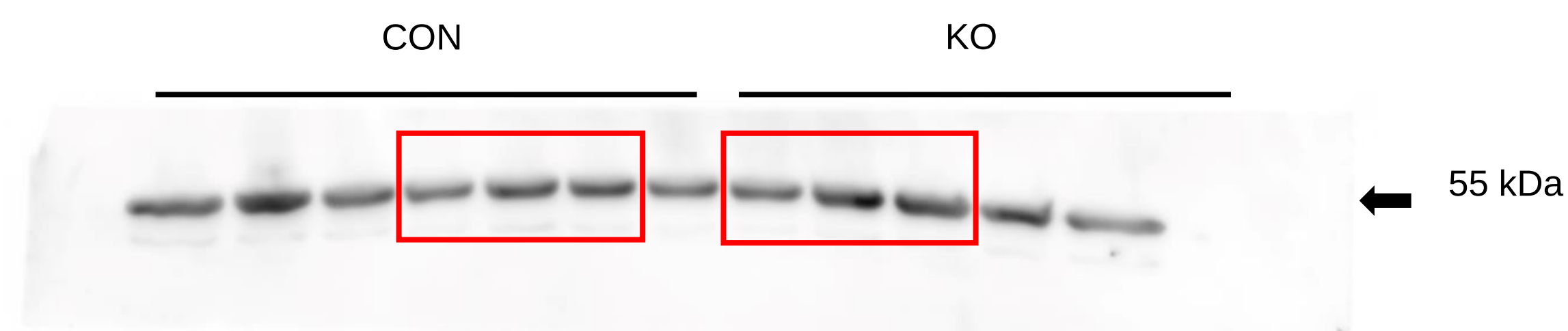

Figure S6b Smmhc

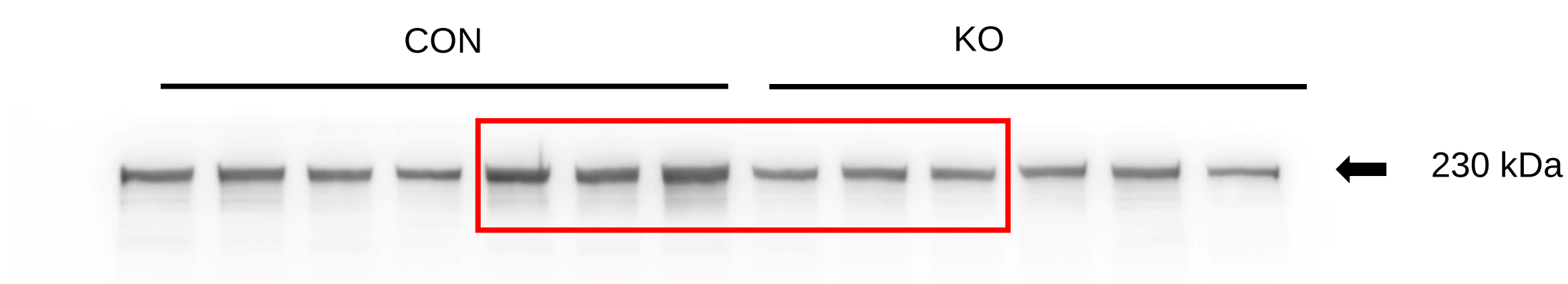

B-tubulin

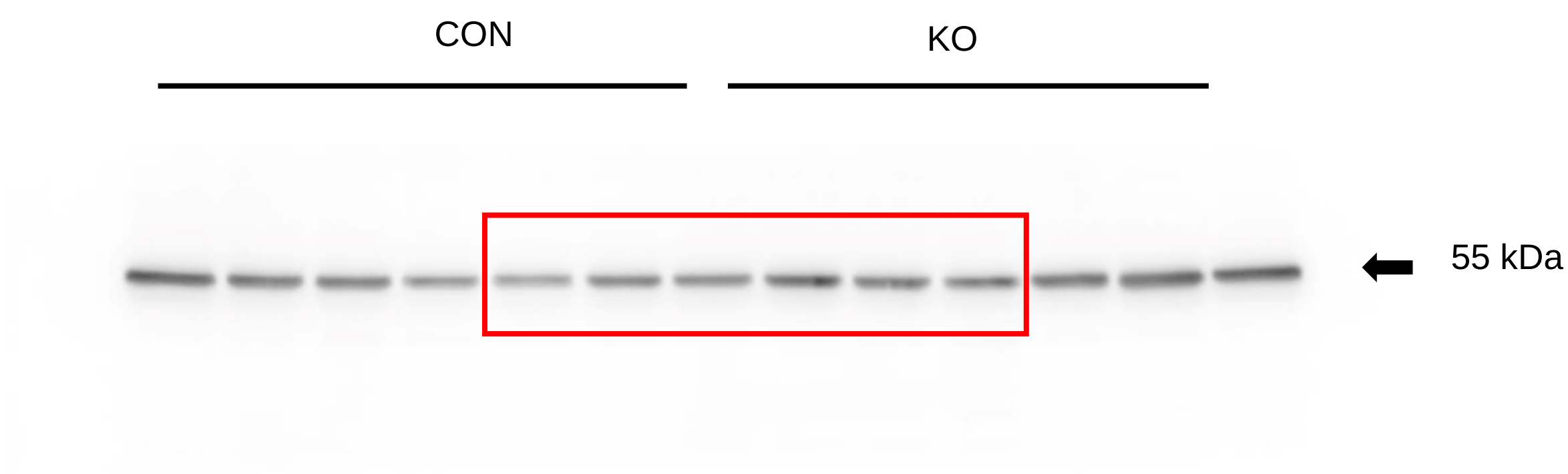

Figure S6c Mlc2

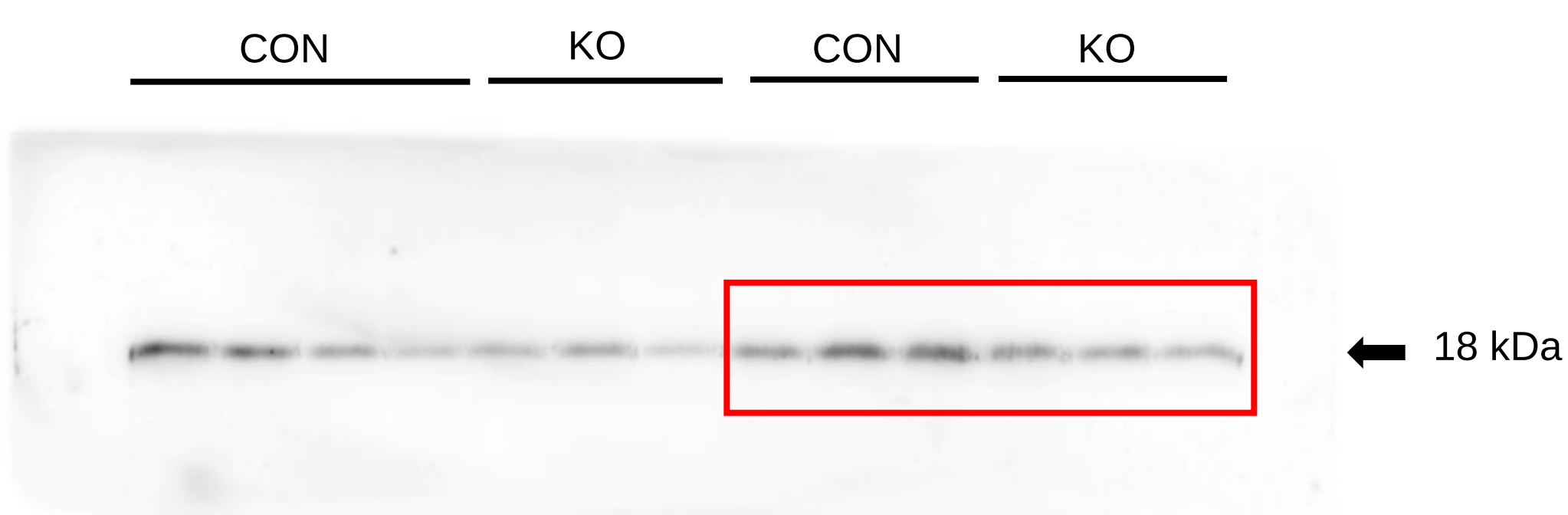

B-tubulin

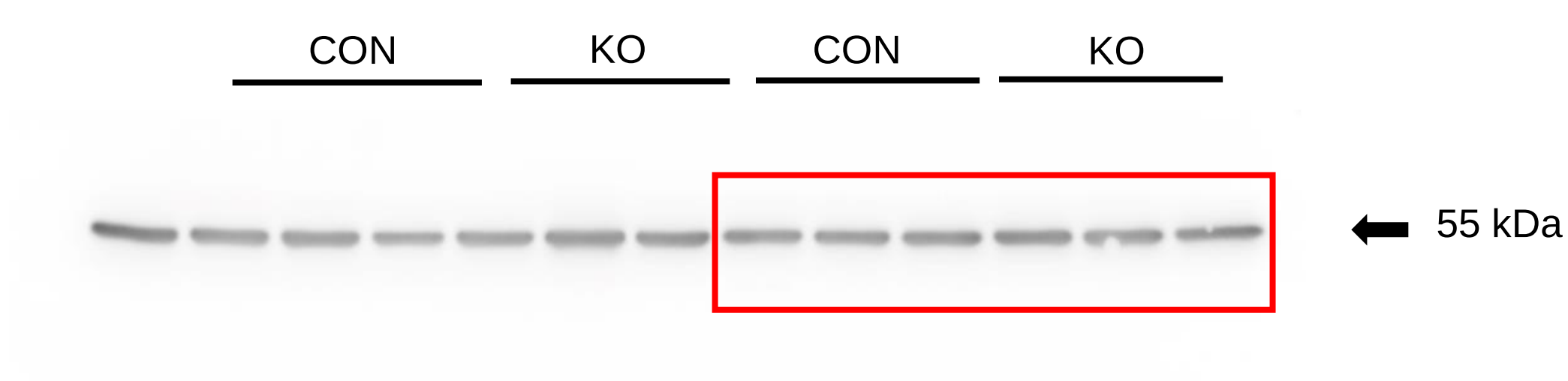

Figure S6d Mlck

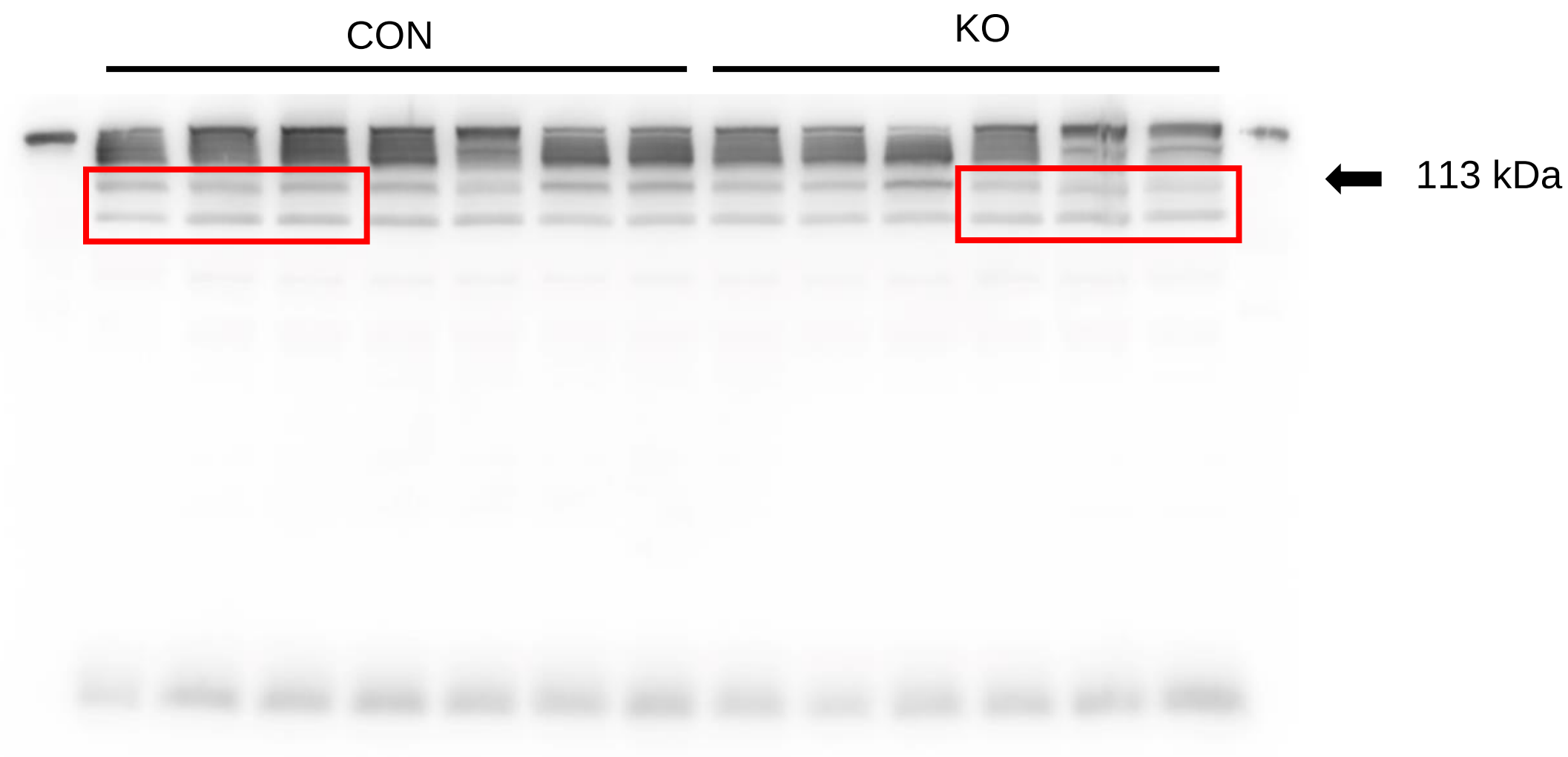

B-tubulin

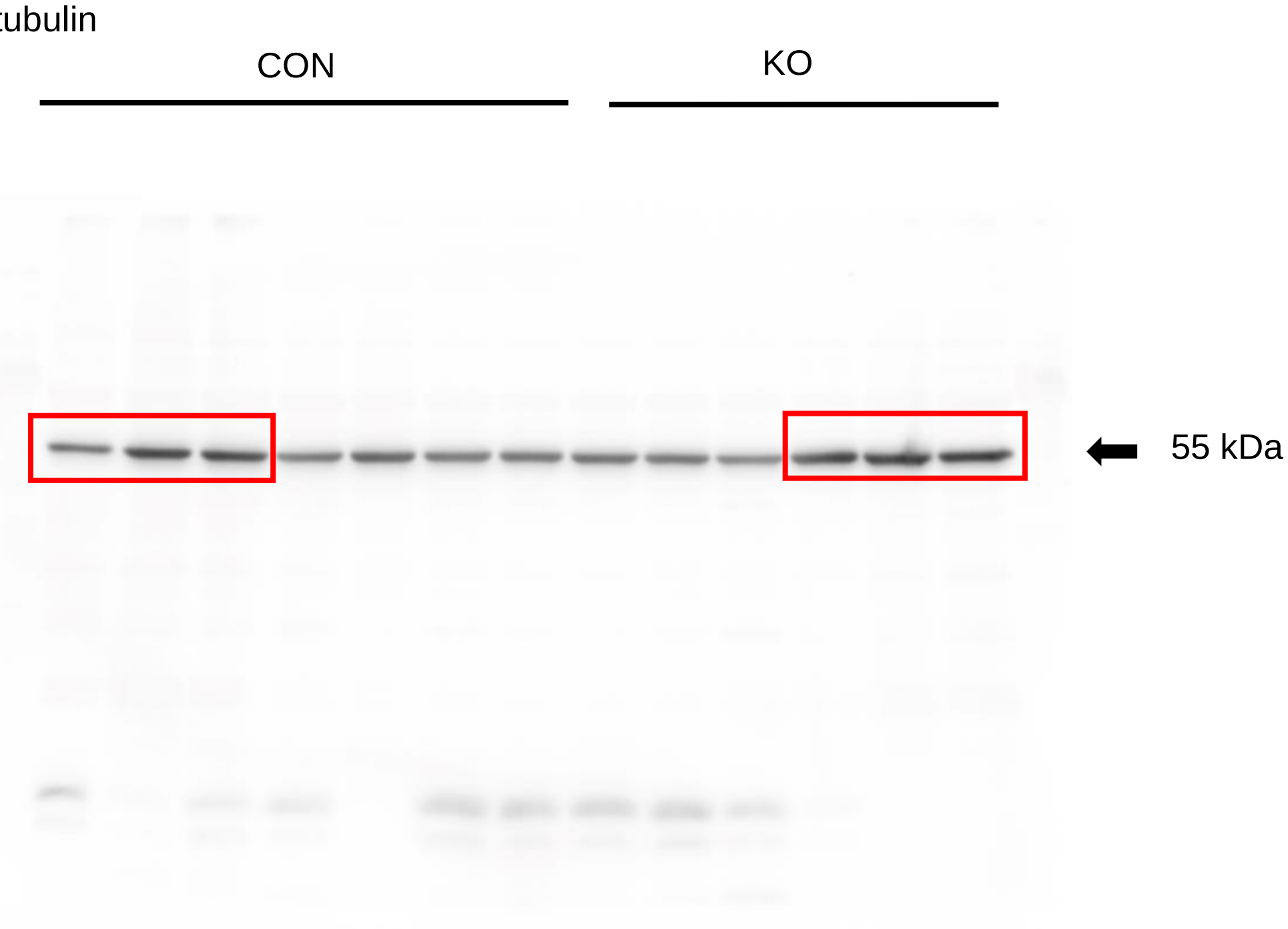

Figure S6e Telokin

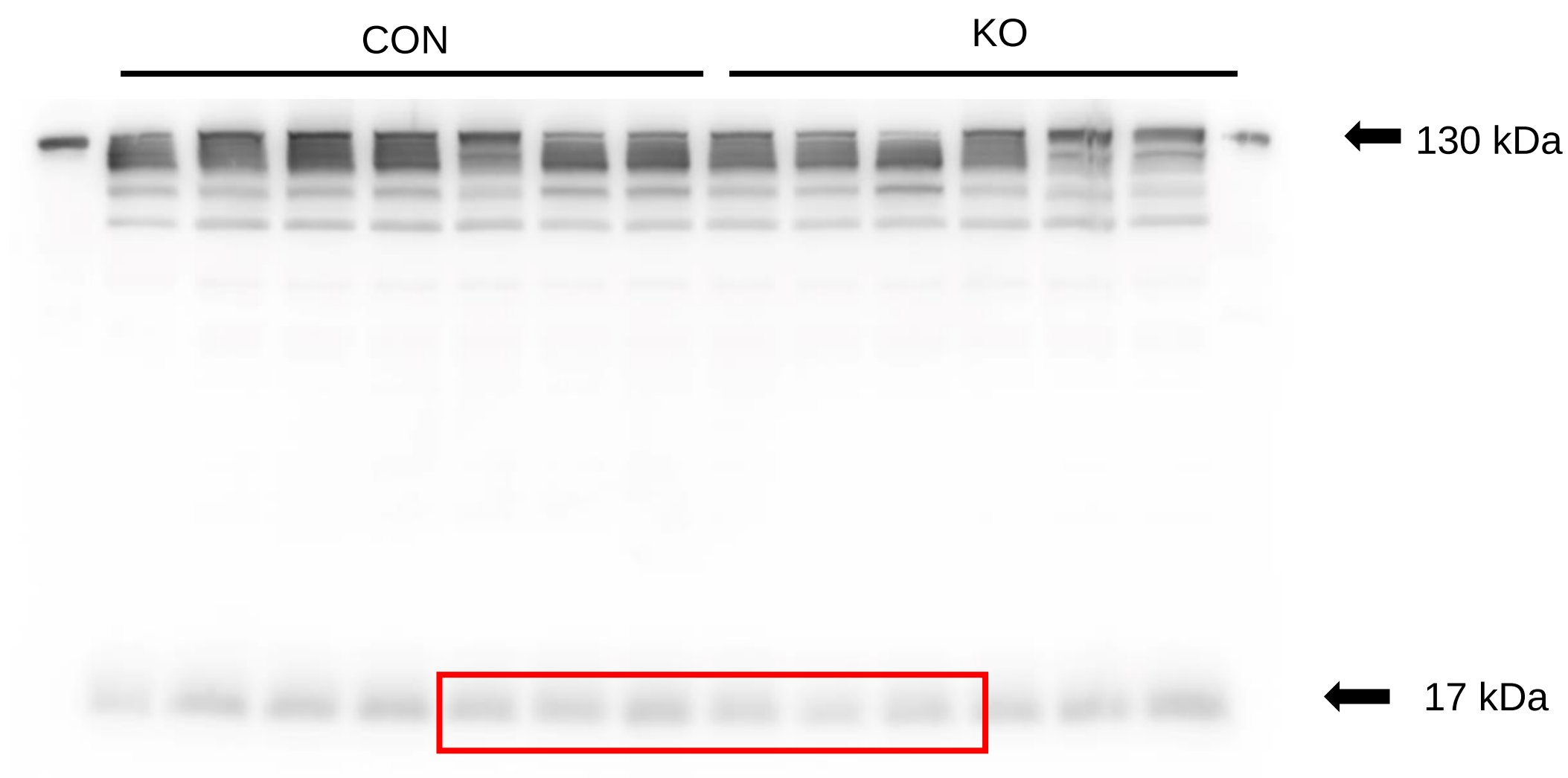

B-tubulin

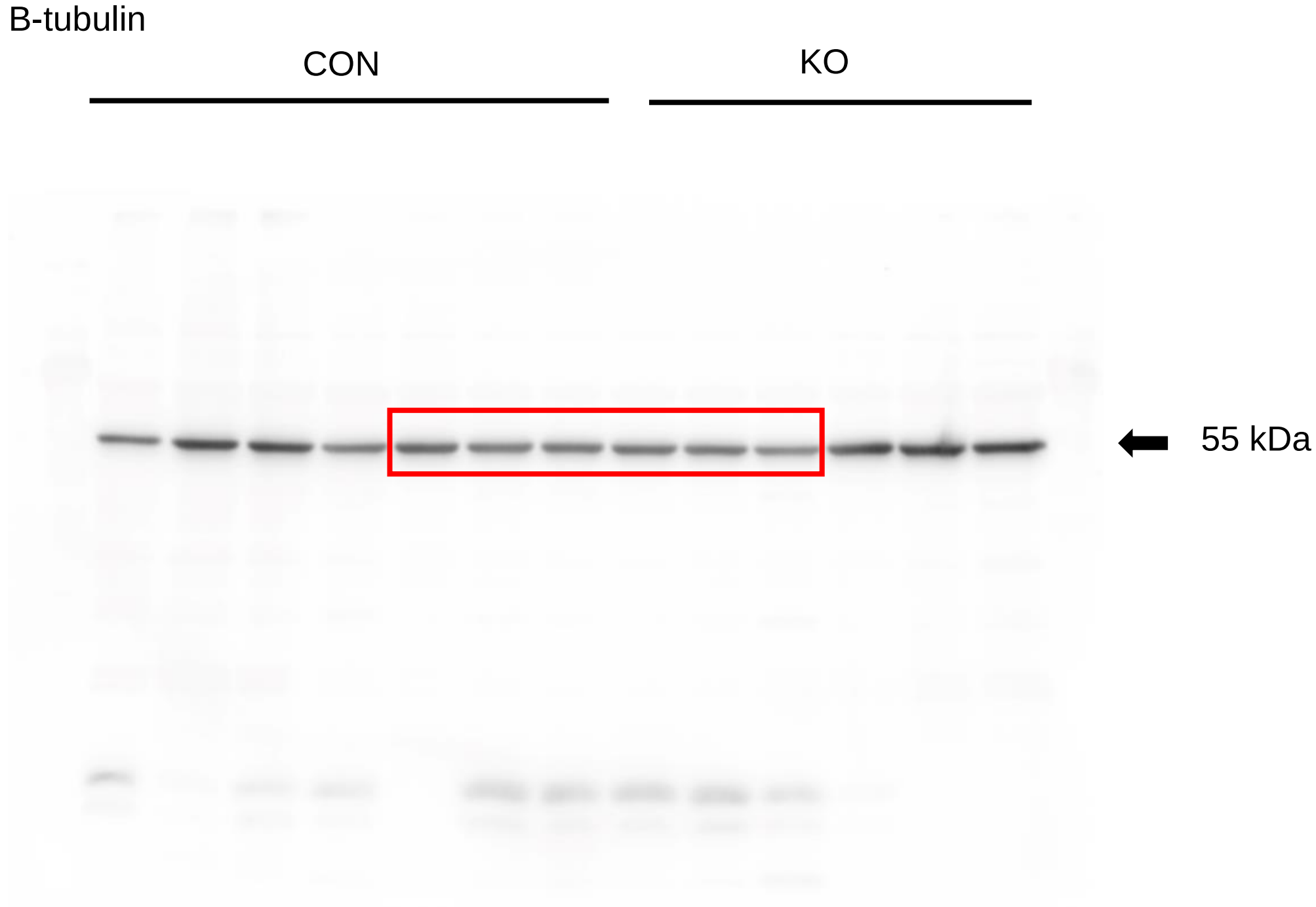

Supplement: Supplementary file 1 [file mmc1.pdf]
